# RNA-Binding Proteins in Cancer: Functional and Therapeutic Perspectives

**DOI:** 10.3390/cancers12092699

**Published:** 2020-09-21

**Authors:** Donghee Kang, Yerim Lee, Jae-Seon Lee

**Affiliations:** 1 Medical Research Center, College of Medicine, Inha University, Incheon 22212, Korea; dongheekang@inha.edu (D.K.); 318067@inha.ac.kr (Y.L.); 2 Department of Molecular Medicine, College of Medicine, Inha University, Incheon 22212, Korea; 3 Program in Biomedical Science & Engineering, Inha University Graduate School, Incheon 22212, Korea

**Keywords:** RNA-binding proteins (RBPs), mechanistic function, cancer phenotype, cancer therapy

## Abstract

**Simple Summary:**

RNA-binding proteins (RBPs) play central roles in regulating posttranscriptional expression of genes. Many of them are known to be deregulated in a wide variety of cancers. Dysregulated RBPs influence the expression levels of target RNAs related to cancer phenotypes, such as proliferation, apoptosis, angiogenesis, senescence, and EMT/invasion/metastasis. Thus, understanding the molecular functions of RBPs and their roles in cancer-related phenotypes can lead to improved therapeutic strategies.

**Abstract:**

RNA-binding proteins (RBPs) crucially regulate gene expression through post-transcriptional regulation, such as by modulating microRNA (miRNA) processing and the alternative splicing, alternative polyadenylation, subcellular localization, stability, and translation of RNAs. More than 1500 RBPs have been identified to date, and many of them are known to be deregulated in cancer. Alterations in the expression and localization of RBPs can influence the expression levels of oncogenes, tumor-suppressor genes, and genome stability-related genes. RBP-mediated gene regulation can lead to diverse cancer-related cellular phenotypes, such as proliferation, apoptosis, angiogenesis, senescence, and epithelial-mesenchymal transition (EMT)/invasion/metastasis. This regulation can also be associated with cancer prognosis. Thus, RBPs can be potential targets for the development of therapeutics for the cancer treatment. In this review, we describe the molecular functions of RBPs, their roles in cancer-related cellular phenotypes, and various approaches that may be used to target RBPs for cancer treatment.

## 1. Introduction

RNA-binding proteins (RBPs) play central roles in regulating gene expression at the post-transcriptional level. Recent advances in screening techniques have led to the identification of more than 1500 RBPs, which account for about 7.5% of all protein-coding genes in the human genome. RBPs can interact with proteins and various classes of RNAs (mRNAs, ncRNAs, tRNAs, snRNAs, snoRNAs, and others) to form ribonucleoprotein (RNP) complexes [1]. RBPs recruit various factors and enzymes, and form different complexes in diverse combinations to modulate the fates and/or functions of target RNAs [2,3]. Conventional RBPs have various impacts on RNA metabolism, such as by regulating microRNA (miRNA) processing and the alternative splicing, alternative polyadenylation, subcellular localization, stability, and translation of RNAs. More than half of the RBPs harbor one or more RNA-binding domains (RBDs), such as the RNA-recognition motif (RRM), K-homology (KH) domain, double strand RNA-binding domain, zinc-finger domains, PAZ domain, and others. Indeed, the RBPs can be classified by their RBDs. The RBPs use these RBDs to form sequence-dependent or structure-specific interactions with target RNAs [4]. Recent studies involving RNA interactome capture and RBP structural analysis have highlighted the nonconventional RBPs, which do not contain any canonical RBD for forming protein-RNA complexes. Numerous nonconventional RBPs have been found to participate in a broad range of biological processes, but their relationships with RNAs have not yet been fully elucidated [2,5].

Many studies have suggested that dysregulated RBPs are associated with various human diseases, including numerous cancers [6]. In cancer, aberrantly expressed RBPs regulate the expression levels of target RNAs related to cancer cell proliferation, apoptosis, angiogenesis, senescence, and epithelial-mesenchymal transition (EMT)/invasion/metastasis. In a broad sense, RBP-mediated regulation ultimately contributes to cancer development and pathology [6,7]. Given that RBPs are critical regulators of cancer, they could be promising targets of cancer therapeutics [6,7]. Thus, improving our understanding of the mechanistic, functional, and pathological roles of RBPs in cancers will contribute to providing therapeutic perspectives for cancer therapy.

In this review, we will discuss various molecular and cellular functions of RBPs in cancer, as well as their clinical implications and potential therapeutic strategies.

## 2. Mechanistic Roles of RBPs in Cancer

### 2.1. miRNA Processing

RBPs act as key regulators of miRNA biogenesis and maturation; they primarily facilitate or inhibit miRNA processing through their effects on canonical proteins, such as Drosha and Dicer. In cancer, alteration of RBP expression is closely linked to the impairment of miRNA processing, which leads to modulation of target mRNAs related to cancer progression and development [8].

LIN28 proteins (LIN28A and LIN28B) are RBPs that contain three RBDs: a cold-shock domain (CSD) and two zinc-knuckle domains (ZKDs). These domains of LIN28 proteins are required for the selective recognition of members of the *let-7* miRNA family and the binding of LIN28 proteins to the terminal loops (TL) of these miRNAs [9]. In the nucleus, LIN28B binds to the TL of pre-*let-7* and inhibits its miRNA processing by negatively regulating microprocessor activity [10]. In contrast, LIN28A plays regulatory roles in the cytoplasm: It recognizes a tetra-nucleotide sequence motif (GGAG) and recruits uridylyl transferase (TUTase) TUT4/7 to pre-*let-7*, and the formed LIN28-TUT4/7 promotes uridylation at the 3′-end of pre-*let-7* to block Dicer processing. Subsequently, the uridylated pre-*let-7* is degraded by the 3′-5′ exoribonuclease, DIS3L2 [11,12,13]. Functionally, LIN28-mediated *let-7* downregulation derepresses the target genes of *let-7*, which include *K-Ras*, *c-Myc*, *HMGA1*, *HMGA2*, *VEGF*, *PDK1*, *Cyclin D*, *BMP4*, *IL-6*, *IGF1R*, and *IGF2BP2.* Eventually, LIN28/*let-7* pathway leads to cancer development and progression-related phenotypes, including proliferation, invasion, metastasis, tumor-promoting inflammation, and angiogenesis [14,15].

KH-type splicing regulatory protein (KHSRP, also known as KSRP) is a component of the Drosha and Dicer complexes. KHSRP binds to the TL of a target miRNA precursor with high affinity and facilitates the maturation of miRNAs, such as *miR-26a*, *miR-26b*, *let-7a*, *miR-23a*, *miR-23b*, *miR-192-5p*, *miR-21*, *miR-130b*, and *miR-301* [16,17,18,19,20,21]. In small cell lung cancer (SCLC), high-level expression of KHSRP promotes *miR-26a* maturation. Increased *miR-26a* binds to the 3′-UTR of *PTEN* to inhibit its expression [17]. In *miR-26* and *let-7a* mediates the translational silencing of *IL-6* and *IL-1α* mRNA by binding to 3′-UTR of *IL-6* and *IL-1α* [20]. In non-small cell lung cancer (NSCLC), KHSRP promotes the maturation of *miR-23a* precursors. Increased *miR-23a* downregulates *EGR3* expression through binding to its 3′-UTR, leading to inhibition of NSCLC mobility [18]. The *miR-192-5p* precursor is subjected to KHSRP-mediated maturation in NMuMg cells (a murine immortalized mammary epithelial cell line). Increased *miR-129-5p* regulates the expression of EMT factors, such as *Fn1*, *Col12a1*, and *Col6a2* [19]. In esophageal squamous cell carcinoma (ESCC), *miR-21*, *miR-130b*, and *miR-301* downregulate their target mRNAs, which include *BMP6*, *PDCD4*, and *TIMP4*, and induces EMT [21]. KHSRP functions as either a tumorigenic or tumor-suppressive protein by regulating miRNA biogenesis [16,17,18,19,20,21].

Heteronuclear ribonucleoprotein A1 (hnRNP A1) harbors two RRMs and has been implicated in miRNA processing [22]. hnRNP A1 acts as an auxiliary factor for the processing of miRNA precursors, such as pri-*miR-18a*. It recognizes two UAG motifs of pri-*miR-18a* (one in the TL and one in the proximal stem region), forms a 1:1 complex with this miRNA, and increases the efficiency of Drosha cleavage by causing relaxation at the stem of the pri-miRNA [22]. In lung cancer, cervical cancer, prostate cancer, gastric cancer, and mesothelioma *miR-18a* downregulates target mRNAs, such as *IRF2*, *PTEN*, *WNK2*, *SOX6*, *STK4*, and *PIAS3*, to induce cancer progression and development. In other cancers, in contrast, *miR-18a* negatively regulates *CDC42* and *SREBP1* to suppress cancer cell proliferation, invasion, and metastasis [23]. Conversely, hnRNP A1 negatively regulates the processing of *let-7a* by binding to the conserved terminal loop of pri-*let-7a-1* to block the interaction of KHSRP, which increases *let-7a* biogenesis [24]. hnRNP A1 and KHSRP play antagonistic roles in the processing of *let-7a* precursors [24].

SMAD, which is a signal transducer of TGF-β and BMP, positively regulates the maturation of *miR-21* and *miR-199a*, which contain the RNA Smad-binding element (R-SBE) [25]. SMAD directly binds to this consensus sequence and recruits RNA helicase p68 (also called DDX5), which is a component of the Drosha microprocessing complex. The interactions of SMAD with pri-*miR-21* and pri-*miR-199a* increase the expression levels of *miR-21* and *miR-199a* by mediating their efficient cleavage by the Drosha complex [25]. Increased *miR-21* negatively regulates the tumor suppressor, *PDCD4*, by binding to its 3′-UTR, and this promotes tumor invasion, intravasation, and metastasis [26].

### 2.2. Alternative Splicing

Alternative splicing is a critical mechanism through which pre-mRNA transcripts are processed to generate multiple mRNA variants with different stabilities and protein-coding potentials. Different splice variants of mRNAs contribute to protein diversity in cancer [27]. Alternative splicing events are controlled by the core spliceosome, which is a large molecular complex composed of small nuclear proteins (snRNPs), polypeptides, and RBPs. Alternative splicing is regulated by cis-acting elements (i.e., enhancers or silencers located in exons or introns) and trans-acting splicing factors, which promote or suppress exon splicing, respectively. RBPs, such as SRSF, hnRNPs, PTB, ESRP, and QKI, bind to cis-regulatory elements and form trans-acting splicing factors to regulate (negatively or positively) the splicing reaction [28]. Compared to normal cells, aberrant patterns of alternative splicing are seen in cancer cells. These alterations can lead to modulation of oncogenes or tumor-suppressive genes, and thereby help drive cancer development and progression [28,29].

Serine/Arginine-rich (SR) family proteins, which represent the best-characterized splicing regulators, have one or two RRMs and a C-terminal arginine (R) and serine (S) amino acid sequence (RS domain); these functional regions participate in RNA recognition and protein–protein interactions to recruit the spliceosome [30]. SR proteins contribute to multiple steps in splicing events, and they are involved in both constitutive and alternative splicing [31]. SRSF1 (also known as SF2/ASF) is known to alter the splicing of the protooncogene *Ron*, the tumor suppressor *BIN1*, the kinases *MNK2* and *S6K1*, and the proapoptotic regulators *Bcl-x*, *Mcl-1*, *CASP2*, *CASP9*, and *ICAD* [32,33,34,35]. SRSF1 is modulated its alternative splicing activity through the interaction with the lncRNA, *MALAT1*. *MALAT1* influences the distribution of SRSF1 at nucleus and nuclear speckles, thereby modulating the levels of phosphorylated SRSF1 [36]. In hepatocellular carcinoma (HCC), *MALAT1*-upregulated SRSF1 enhances the isoforms of the antiapoptotic gene *BIM* and the oncogenes *S6K1* and *TEAD1*, leading to cancer cell proliferation, survival, and tumorigenesis [37]. Moreover, in glioblastoma, SRSF1 physically associates with *circRNA SMARCAS5* to regulate the alternative splicing of *VEGF*, thereby affecting angiogenesis [38]. SRSF3 (also known as SRp20) is the smallest member of the SR protein family; it is involved in the alternative splicing of *FoxM1* to generate the *FoxM1a*, *b*, and *c* isoforms. Conversely, SRSF3 overexpression increases the *FoxM1b* and *1c* isoforms and results in upregulation of the FoxM1 targets, PLK1 and CDC25B [39]. In colon cancer cells, SRSF3 regulates the alternative splicing of *HIPK2*. Research revealed that SRSF3 depletion promotes skipping of 81 5′ nucleotides (27 amino acids) from exon 8 of *HIPK2*. This generates *HIPK2* Δ*e8*, which has no binding site for SIAH1 ubiquitin ligases, and is thus protected from proteasomal digestion [40]. Transcriptomic profiling of SRSF3-mediated altered splicing showed that SRSF3 binds to the CA(G/C/A)CC(C/A) sequence of the exonic splicing enhancer that is found in target genes, such as those encoding *ETV1* and *NDE1* [41]. In contrast to the above-described situation in colon cancer cells, SRSF3 is reduced or mislocalized in hepatocellular carcinoma. SRSF3 affects the alternative splicing of genes related to metabolism (*HNF1a*, *ERN1*, *HMGCS1*, *DHCR7*, and *SCAP*) or EMT (*LIFR*, *Epb4.1l5*, *Myo1b*, *CTNND1*, *GIT2*, and *SLK*) [42,43]. In colorectal cancer (CRC), the SRSP (splicing regulatory small protein) encoded by the lncRNA *Loc90024* interacts with SRSF3 and promotes its binding to *SP4*. SRSF3-SRSP mediates the alternative splicing of *SP4* and generates cancerous *SP4* isoforms, leading to tumorigenesis and metastasis [44]. SRSF6 (formerly SRp55) is upregulated in a subset of tumors and is critical for tumor growth, initiation, and maintenance [45]. SRSF6 has been shown to regulate the splicing of one oncogene (*INSR*) and tumor suppressor genes (*DLG1* and *MKNK2*), modulating their oncogenic and tumor-suppressive isoforms [45]. SPF45, also known as RBM17, is a component of the spliceosome and associates with the lncRNA *Saf*. In erythroleukemia and cervical carcinoma cells, SPF45-*Saf* binds to the *Fas* pre-mRNA and modulates alternative splicing of *Fas*; this increases soluble Fas, which eventually interacts with Fas ligand to protect cells from apoptosis [46].

hnRNP A/B family members, such as hnRNP A1 and hnRNP A2/B1, regulate alternative splicing by acting as antagonists of the SR proteins [47]. hnRNP A1 has two RBP domains and one arginine-glycine-rich (RGG) domain, and recognizes the UAGGG(A/U) sequence. hnRNP A2/B1 has a structure similar to that of hnRNP A1, and binds to (UUAGGG)_N_ [47]. hnRNP A1 and hnRNP A2/B1 regulate the alternative splicing of the glycolytic enzyme, *PKM2*, and increase the *PKM2/PKM1* ratio in cancer cells [48,49]. hnRNP A1 is overexpressed in hepatocellular carcinoma (HCC); this increases the level of *CD44v6*, which promotes invasiveness in HCC and is associated with a poor prognosis for HCC patients [50]. In glioblastoma, hnRNP A2/B1 functions as a proto-oncogene. This hnRNP affects alternative splicing and increases the expression of oncogenic isoforms of genes related to tumor suppressors (*BIN1* and *WWOX*), anti-apoptotic proteins (*CFLAR* and *CASP9*), and a proto-oncogene (*MST1R*) [51]. hnRNP M controls isoform switching from *CD44* variable (*CD44v*) to *CD44* standard (*CD44s*) [52]. Upregulated hnRNP H generates a constitutively active isoform of the tyrosine kinase receptor, *MST1R* [53].

Polypyrimidine tract-binding protein (PTB, also known as hnRNP I) plays a role as a splicing repressor and preferentially interacts with pyrimidine-rich sequences and modulates cancer-relevant alternative splicing events, leading to exon skipping or inclusion [54]. In glioblastoma, PTB binds to the ISS element in the intron upstream of the *FGFR-1α* exon; it mediates skipping of the α exon to produce the *FGFR-1β* isoform [55]. The switch from *FGFR-1α* to *FGFR-1β* contributes to the malignant progression of astrocytic tumors [56]. *USP5* is subjected to PTB-mediated alternative splicing. High-level expression of PTB generates *USP5* isoform 2 and inhibits the production of *USP5* isoform 1 [57]. In addition, PTB and hnRNP A1/A2 cooperate to regulate an alternative splicing event of *PKM2*; they bind to the ISS element of *PKM* and thereby increase the level of *PKM2* [48,49].

Epithelial splicing regulatory protein 1 (ESRP1) and ESRP2 (also known as RBM35 and RBM35B, respectively) are splicing factors that have closely related structures and are specifically expressed in epithelial cells [58]. ESRP1 and ESRP2 regulate alternative splicing switching in *FGFR2*, *CD44*, *CTNND1*, and *ENAH*. Moreover, ESRP1 and ESRP2 promote the splicing of numerous genes related to cytoskeletal dynamics, cell motility, cell-cell junctions, and EMT [58,59,60].

RBM3, which is known as a proto-oncogene, is a member of the glycine-rich RBP family [61]. In HCC, upregulated RBM3 induces biogenesis of *SCD-circRNA*, leading to cell proliferation [62].

Src-associated in mitosis of 68 kDa (SAM68) is a member of the signal transduction and activation of RNA (STAR) protein family [63]. SAM68 is primarily localized in the nucleus and regulates alternative splicing. SAM68 is a target of ERK and its serine-threonine is phosphorylated in response to RAS/ERK signaling. Phosphorylated SAM68 induces the inclusion of the v5 exon of *CD44* in a protein modification-dependent fashion [64]. In prostate cancer, the phosphorylation of SAM68 by signal transduction pathways increases its binding affinity to the proximal region of *Cyclin D1* intron 4. SAM68 enhances alternative splicing of *Cyclin D1* and inhibits the recruitment of U1-70K to its target genes, generating *Cyclin D1b* [65]. Meanwhile, SAM68 phosphorylated through Src-like kinases alters the splicing of *Bcl-x*, which encodes two splicing variants, anti-apoptotic *Bcl-xL* and pro-apoptotic *Bcl-xS* [66]. Importantly, SAM68 is involved in cancer-relevant splicing in a protein modification-dependent manner [64,65,66].

Quaking (QKI), a member of the STAR family, is involved in the biogenesis of circRNAs. During EMT, QKI binds to the intron of pre-mRNAs and promotes the formation of circRNAs, such as *SMARCAS5*, *POLE2*, *OXNAD1*, *SHPRH*, *SMAD2*, *ATXN2*, *DOCK1*, and *GNB1*. These EMT-associated circRNAs may regulate the migration, invasion, and metastasis of cancer cells [67].

### 2.3. Alternative Polyadenylation

Alternative polyadenylation is a critical process to generate mature RNA transcripts. Alternative polyadenylation occurs within the 3′-UTR of mRNAs and generates different length 3′-UTRs by 3′-end cleavage and polyadenylation (CPA). CPA is carried out by multimeric protein complexes, such as the cleavage and polyadenylation specificity factors (CPSFs), the cleavage stimulation factors (CSTF), and the mammalian cleavage factor complexes and II (CFIm and CFIIm). The 3′-UTR of an mRNA is critical for its maturation, stability, nucleo-cytoplasmic localization, and translation [68]. RBPs can also regulate the CPA of target mRNAs by either recruiting or competing with the polyadenylation machinery proteins [68].

The cytoplasmic polyadenylation element binding proteins (CPEBs), CPEB1-4, regulate the poly(A) tail length of mRNAs with a cytoplasmic polyadenylation element (CPE) by recruiting the translational repression or cytoplasmic polyadenylation factors. The CPEB-family proteins have C-terminal regions containing two RRMs and two zinc-finger-like motifs, and a variable N-terminal region [69]. CPEB1 can alter the gene expression profile by shortening or lengthening the 3′-UTRs of target mRNAs. The loss of CPEB1 results in poly(A) tail lengthening and increased translation of the mRNA for *MMP9* in breast cancer cells [70]. CPEB1 and CPEB2 mediate the polyadenylation of the *HIF-1α* mRNA by binding to its 3′-UTR. Increased *HIF-1α* polyadenylation affects its protein levels [71]. CPEB2 and CPEB3 are less well studied in terms of their roles in cancer, and future work is needed. In pancreatic ductal adenocarcinoma (PDA), overexpression of CPEB4 promotes poly(A) elongation and translational activation for the mRNA encoding tPA, which is a key regulator of PDA malignancy. *tPA* with a longer 3′-UTR has a higher translation efficiency, leading to high-level expression in PDA [72]. CPEB4 is highly expressed in the early stage of melanoma progression; it can control the polyadenylation of the melanoma drivers, *MITF* and *RAB7A*, through binding to their 3′-UTRs. Depletion of CPEB4 decreases the protein levels of MIFT and RAB7A through the shortening of their poly (A) tails [73]. CPEB1 and CPEB4 regulate the length and polyadenylation of the *VEGF* 3′-UTR in the nucleus and cytoplasm, respectively. The *VEGF* 3′-UTR harbors several CPE elements and alternative polyadenylation sites (PASs). In the nucleus, CPEB1 promotes alternative 3′-UTR processing of *VEGF* and increases the level of *VEGF* mRNAs with shorter 3′-UTRs that lack translation inhibitory elements, such as AU-rich elements (AREs) and miRNA-binding sites, and thus are less prone to degradation. CPEB1 also upregulates CPEB4 expression through alternative processing of the pre-mRNA. Activated CPEB4 contributes to positively regulating cytoplasmic VEGF expression through polyadenylation of its mRNAs [74].

### 2.4. RNA Localization

The subcellular localization of an mRNA or lncRNA is an important factor in the regulation of its stability and translation, and cancer-related RBPs often bind to RNAs to coordinate where they are localized and translated [75,76].

Among the CPEB family members, CPEB1 regulates the localization of *ZO-1* mRNA, which encodes a critical tight junction component. The apical co-localization of CPEB1 with the *ZO-1* mRNA is consistent with its role in RNA localization. When CPEB1 is depleted, the *ZO-1* mRNA is randomly distributed and central cavity formation is disrupted, resulting in loss of epithelial cell polarity [77]. This impairment of cell polarity is linked to the metastatic potential, and CPEB1 depletion leads to alterations in EMT-related genes [70].

IGF2BP1 (also called IMP1/ZBP1) controls the subcellular localizations of the mRNAs encoding *β-actin*, *E-cadherin*, *α-actinin*, and *Arp-16* (a component of the Arp 2/3 complex) [78,79]. IGF2BP1 binds to the zip-code of the *β-actin* mRNA via its COOH-terminal KH domains and localizes the *β-actin* mRNA to the cell periphery [78]. In breast carcinoma cells, IGF2BP1 promotes the localization of *E-cadherin*, *α-actinin*, and *Arp-16* at cell–cell contacts. IGF2BP1 also regulates the localizations of mRNAs related to cell motility and focal adhesions to impact cancer invasion and metastasis [78,79]. IGF2BP2 (also called IMP2) binds to oxidative phosphorylation (OXPHOS)-related mRNAs, such as *NDUFS3* and *COX7b*, and facilitates their localization to mitochondrial polysomes. The delivery of these mRNAs to mitochondria induces their translation and insertion into mitochondrial membranes, contributing to the assembly of complexes I and IV [80].

HuR and GRSF1 regulate the shuttling of the lncRNA *RMRP* from the nucleus to mitochondria. HuR, which is predominantly localized in the nucleus, promotes the nuclear export of lncRNA *RMRP* in a CRM1-dependent manner. After lncRNA *RMRP* is transported into mitochondria, GRSF1 is involved in the retention of lncRNA *RMRP* [81].

Another RBP, hnRNP K, can directly associate with the lncRNA *MALAT1* and regulates its nuclear retention. hnRNP K binds to a sequence derived from Alu repeats and short interspersed nuclear element (SINEB1), and increases the distribution of *MALAT1* at the nucleus [82,83].

### 2.5. RNA Stability

The stability of an RNA is determined by its 5′ -terminal 7-methylguanosine (m^7^G) cap and 3′ poly(A) tail. These two stability determinants protect mRNAs from decay and promote translational initiation [84]. mRNA decay is mediated through several pathways, including deadenylation of the poly(A) tail, removal of the 5′ -terminal m^7^G cap (decapping), 5′→3′ exonucleolytic decay, and exosome-mediated 3′→5′ degradation. mRNAs targeted for decay are shuttled to cytoplasmic foci, such as processing bodies (P-bodies) or stress granules [84]. RBPs, such as AUF1, HuR, TTP, IGF2BP family proteins, and Wig1, can stabilize or destabilize specific target mRNAs and lncRNAs in cancer [85].

AU-rich element RNA-binding protein 1 (AUF1; also known as heterogeneous nuclear ribonucleoprotein D, or hnRNP D) comprises four proteins: p37^AUF1^, p40^AUF1^, p42^AUF1^, and p45^AUF1^. AUF1 binds to the AREs of mRNAs and primarily promotes mRNA decay via ARE-mediated decay (AMD) [86]. In NSCLC cells, p45^AUF1^ is increased by the chemotherapeutic agent, prostaglandin A_2_, and this induction increases *Cyclin D1* mRNA turnover by binding the *Cyclin D1* 3′-UTR [87]. AUF1 also negatively affects the stability of *p21* and *Cyclin D1* by directly binding to their 3′-UTRs. Knockdown of AUF1 was found to increase the stabilities and expression levels of *p21* and *Cyclin D1* [88]. AUF1 promotes the destabilization and decay of the *p16* mRNA by binding to a stem-loop structure localized within its 3′-UTR [89]. AUF1 and HuR share the *Cyclin D1*, *p21*, and *p16* mRNAs as common targets. They co-occupy the *p21* and *Cyclin D1* mRNAs at non-overlapping sites or competitively bind these mRNAs at common sites [88]. For decay of the *p16* mRNA, meanwhile, AUF1 can function as a co-factor of HuR; the co-factors interdependently interact with a secondary structure within the *p16* 3′-UTR to destabilize the *p16* mRNA [89]. Other cell cycle-regulatory proteins, such as *p27* and *pRB*, are targets of AUF1, which negatively regulates their mRNA stabilities [90]. AUF1 also mediates the destabilization of apoptosis regulators (*Bax*, *Bcl-2*, *Gadd45a*, and *CASP2*), metastasis regulators (*MMP9* and *FGF9*), inflammatory factors (*GM-CSF*, *IL-6*, and *NOS*), and DNA repair, replication, and replication regulators (*Fos*, *TYMS*, and *JunD*) [91]. Moreover, AUF1 interacts with and destabilizes the cancer-associated lncRNA *NEAT1* [92].

HuR, a member of the Hu family of RNA-binding proteins, governs the stability of target mRNAs by binding to AREs in their 3′-UTRs. HuR-mediated mRNA stabilization depends on the subcellular localization of HuR, which is translocated into the cytoplasm in response to different stresses and stimuli, including DNA damage, oxidative stress, and chemical compound treatment. The increased cytoplasmic HuR binds to the 3′-UTRs of mRNAs related to oncogenes, cell cycle regulators, inflammation, and apoptosis. For example, HuR stabilizes the mRNAs for Cyclins (*A*, *B1*, *D1*, and *E*), *IL-8*, *MMP9*, *HIF-1α*, *VEGF*, *SIRT1*, and *Snail*, thereby elevating their proteins levels. In contrast, mRNA levels of *c-Myc*, *Wnt5a*, and *p27* are downregulated by HuR [93]. Moreover, HuR cooperates with RBPs or miRNAs to regulate mRNA stability. In CRC, HuR interferes with the binding of *miR-16* to the *COX-2* mRNA, thereby increasing *COX-2* expression [94]. HuR is involved in stabilizing various lncRNAs, such as *NEAT1*, *lncRNA-HGBC*, and *OIP5-AS1* [95,96,97]. In ovarian cancer, HuR stabilizes *NEAT1* by inhibiting its binding with *miR-124-3p*, and upregulation of *NEAT1* in ovarian cancer induces cancer cell proliferation and invasion [95]. In gallbladder cancer, *lncRNA-HGBC* stability is increased by HuR. Stabilized *lncRNA-HGCB* functions as an miRNA sponge to prevent *miR-502-3p* from binding to the target gene *SET*. This leads to activation of the *SET*-AKT pathway, which supports cancer cell proliferation and metastasis [96]. HuR also interacts with and stabilizes the lncRNA *OIP5-AS1*. This interaction prevents the binding of HuR to target mRNAs, such as *Cyclin A2*, *Cyclin D1*, *SIRT1*, *VHL*, *TP53*, and *WEE1*, and thereby inhibits the proliferation of cervical carcinoma cells [97]. In contrast, HuR associates with the lncRNAs *LincRNA-p21* and *HOTAIR* to facilitate *let-7*/Ago2-mediated decay of those lncRNAs [98,99].

Tristetraprolin (TTP) is a cysteine-cysteine-cysteine-histidine (CCCH) zinc-finger protein that recognizes AREs in the 3′-UTRs of target mRNAs. The binding of TTP to the 3′-UTR of an mRNA promotes its decay by shortening its poly (A) tail [100]. In the cytoplasm, TTP associates with the Ccr4-Not1 complex and recruits Caf1 deadenylase, leading to hydrolysis of the poly(A) tail of target mRNAs [101]. TTP also mediates decapping of target mRNAs by interacting with decapping complex, which includes Hedls, mRNA-decapping enzyme (Dcp) 1, Dcp2, enhancer of mRNA-decapping protein 3 (Edc3), and the RNA helicase Rck/p54 [102]. TTP mainly functions as a tumor suppressor by targeting mRNAs encoding proteins related to the cell cycle (*Cyclin B1* and *D1*), cell death and proliferation (*Bcl-2* and *cIAP*), angiogenesis (*VEGF*), and EMT (*Snail*, *Twist1*, *ZEB1*, *SOX9*, *MACC1*, *MMP2*, *MMP9*, and *IL-6*) [103]. Recent studies revealed that *PD-L1* is a novel target of TTP in several types of cancer cell. In gastric cancer cells, overexpressed TTP negatively regulates the mRNA stability of *PD-L1* and thereby contributes to the induction of anti-tumor immunity [104].

IGF2BP 1, 2, and 3 (also known as IMP, CRD-BP, VICKZ, ZBP, Vg1RBP/Vera, or KOC) represent a conserved RNA-binding protein family whose members have two N-terminal RRMs and four C-terminal KH domains. The IGF2BP family proteins are primarily localized in the cytoplasm; they promote target mRNA stability by interfering with endonuclease cleavage or miRNA binding [105,106,107,108,109,110]. IGF2BP1 associates with the coding region determinant (CRD) and 3′-UTR of the *β-TrCP1* and *c-Myc* mRNAs, and shields these mRNAs from endonucleolytic cleavage to prevent their degradation [105,106,107]. IGF2BP1 stabilizes the *CD44* mRNA through binding to its 3′-UTR, and thereby contributes to cellular adhesion and invasion during cancer development and formation [107]. IGF2BP1 also binds to and stabilizes the *β-TrCP1* mRNA by interfering with its *miR-183*-dependent interaction with Ago2 [108]. In contrast, IGF2BP1 mediates the destabilization of the liver cancer-associated lncRNA *HULC* by recruiting the Ccr4-Not1 complex in HCC [111]. IGF2BP2 shields the *RAF1* mRNA from *miR-195*-mediated degradation [109]. IGF2BP3 protects *HMGA2* from *let-7a*-directed mRNA decay [110]. Recent studies revealed that IGF2BPs are readers of *N*^6^-methyladenosine (m^6^A, methylation of adenosine at position 6); they recognize the consensus GG(m^6^A)C sequence and promote target mRNA stability [112]. In cancer cells, IGF2BP increases the stability and storage of the *Myc* mRNA [112].

Wig1 (also called ZMAT3) is a p53-target gene and can interact with double-stranded RNAs through its zinc-finger domains [113]. Wig1 regulates the stabilities of the *p53*, *p21*, and *ACOT7* mRNAs by interacting with other proteins, such as hnRNP A2/B1 and Ago2 [114,115,116,117]. Wig1 binds to the ARE in the *p53* 3′-UTR and thereby protects the mRNA from deadenylation by hnRNP A2/B1 [114,115]. Wig1 mediates miRNA-mediated mRNA decay through an association with Ago2, which is a fundamental component of RNA-induced silencing complex (RISC). Wig1 preferentially binds to a stem-loop structure in the 3′-UTRs of the *p21* and *ACOT7* mRNAs; this facilitates the recruitment of Ago2, increasing the ability of RISC to access the *p21* and *ACOT7* mRNAs. The Wig1- and Ago2-containing RISC thus triggers the miRNA-mediated decay of the *p21* and *ACOT7* mRNAs [116,117].

### 2.6. Translational Regulation

Translational control is a critical means by which gene expression is tuned and regulated in cancer. RBPs, which form the dynamic component of the ribonucleoprotein (RNP) complex, are involved in diverse steps of translation, such as initiation, elongation, and termination. Most of the involved RBPs bind to the 5′-or 3′-UTR with different RBP-binding capacities, leading to different translation efficiencies [118].

Eukaryotic translation initiation factor 4E (eIF4E) is a component of the eIF4F translation initiation complex. eIF4E interacts with eIF4A (an RNA helicase) and eIF4G (a scaffolding molecule), and binds to the 5′ -terminal m^7^G cap of mRNAs. After mRNAs are unwound, ribosomes are recruited to mRNAs and translation is initiated. eIF4E is overexpressed in cancer and exerts oncogenic potential by regulating mRNAs related to proliferation (*c-Myc*, *CDK2*, and *Cyclin D1*), metastasis (*MMP9* and *heparanase*), loss of apoptosis (*Mcl-1*, *Bcl-2*, and *survivin*), and angiogenesis (*VEGF* and *FGF2*) [119].

HuR regulates the translation of target mRNAs containing ARE in their 3′-UTRs. HuR enhances the translation of the transcripts encoding *ProTα*, *p53*, and *MSI1* by binding to their 3′-UTRs in an miRNA-independent fashion [120,121]. HuR increases the cytoplasmic abundance and translation of the *ProTα* mRNA by binding to its 3′-UTR, and an increased level of cytoplasmic *ProTα* mRNA has been correlated with anti-apoptotic effects in cancer cells [88]. An interaction between HuR and the *p53* 3′-UTR has been identified in ultraviolet C (UVC)-treated colon cancer cells. After UVC irradiation, the association of HuR with the *p53* 3′-UTR is increased, as is the translation of p53 [120]. HuR positively regulates the *MSI1* mRNA and its translation in glioblastoma [121]. HuR and PTB (also known as hnRNP I) associate with the *HIF-1α* 5′ - and 3′-UTRs, respectively. These RBPs cooperatively increase *HIF-1α* translation in hypoxia [122]. In addition, HuR can negatively regulate mRNA translation in an miRNA-dependent manner. For example, HuR recruits *miR-19* to the *RhoB* 3′-UTR, and thereby represses *RhoB* translation [123].

Musashi (MSI) proteins (MSI1 and MSI2) are members of the class A/B heterogeneous nuclear ribonucleoprotein (hnRNP) family. All MSI proteins have two RRMs (RRM1 and RRM2) for interacting with RNA. MSI1 and MSI2 can positively or negatively regulate mRNA translation in cancer. In breast and brain cancers, MSI1 binds to and represses the mRNA of *NF-YA*, which is a transcription factor that directs the expression of proteasome subunits. The decreased NF-YA protein level leads to downregulation of 26S proteasome subunit expressions [124]. MSI2 induces the translation of the *HOXA9*, *Myc*, and *IKZF2* mRNAs, but represses that of the *p27* mRNA [125,126]. In pancreatic adenocarcinoma, MSI1 and MSI2 positively regulate the translation of *BRD4*, *c-Met*, and *HMGA2* [127]. Meanwhile, both MSI proteins suppress the translation of *p21* and *NUMB* [126,127].

RBPs can contribute to translational control by recognizing structural RNA elements, such as the internal ribosome entry site (IRES) and TGF-β-activated-translation (BAT) element [128,129,130,131]. The IRES is a structural RNA element in the mRNA 5′-UTR that promote selective mRNA translation in a cap-independent manner. For example, HuR binds to the IRES of the *XIAP* mRNA and enhances its translation by increasing its recruitment into the polysome. The HuR-mediated induction of *XIAP* contributes to cytoprotection against apoptosis-inducing agents [128]. The BAT element is a stem-loop region with an asymmetrical bulge; it is located in the mRNA 3′-UTR. hnRNP E1 forms an RNP complex with eukaryotic elongation factor-1 A1 (eEF1A1) by binding to a 33-nucleotide BAT element in the 3′-UTR of the mRNAs encoding *Dab2* and *ILEI*. hnRNP E1 blocks the release of eEF1A1 from the ribosomal A site, which inhibits the translational elongation of *Dab2* and *ILEI*. This translational silencing regulates EMT in tumorigenesis and metastatic progression [129,130]. PDCD4 acts as a translational repressor by inhibiting the helicase activity of eIF4A. However, in eIF4A-independent manner, PDCD4 mediates translational repression by binding to a secondary structure in the coding region of target mRNAs. PDCD4 recognizes a secondary structure in the coding region of the *A-Myb* proto-oncogene, and inhibits its translational elongation [131]. Wig1 also interacts with a secondary structure of the *ACOT7* mRNA and represses its translation. Wig1 bound to the *ACOT7* mRNA forms a complex with RISC components (Ago2 and GW182) and the translation initiation factor, eIF5B, to inhibit translation of the *ACOT7* mRNA [117].

**Figure 1:**
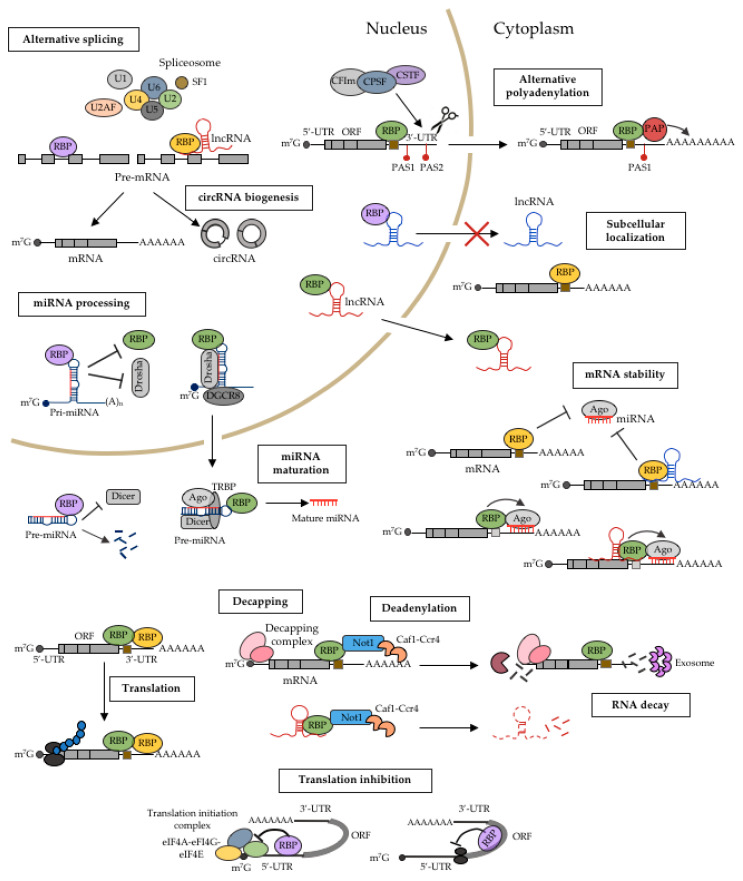
Mechanistic roles of RBPs in the posttranscriptional regulation of gene expression in cancer. RBPs are key regulators of gene expression at the post-transcriptional level. They can determine the fate of an RNA by regulating various events, including their miRNA-mediated processing, alternative splicing, alternative polyadenylation, subcellular localization, stability, and translation. Schematic diagrams of the functions of RBPs in these mechanisms are shown.

Herein, we describe the mechanistic roles of RBPs in cancer. Schematic diagrams of representative RBPs are shown in Figure 1.

## 3. Implications of RBPs in Cancer Phenotypes

**Figure 2:**
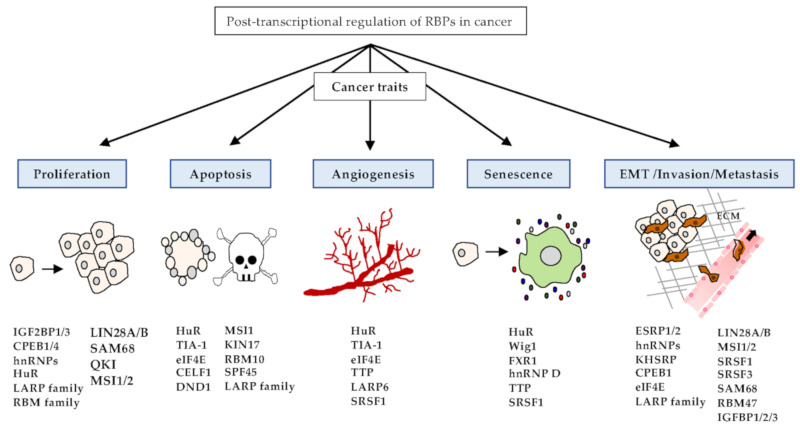
Functions of RBPs in cancer traits. RBPs play pivotal roles in the embodiment of various cancer traits, such as proliferation, apoptosis, angiogenesis, senescence, and EMT/invasion/metastasis. Representative RBPs of cancer traits are listed in the schematic diagram.

Cancer has common features, including abnormal cell growth and division. There are many causes of cancer development, including genetic and environmental factors [132,133]. Among the various causes, cancer development can be triggered by genetic alterations of RBPs [3]. RBPs can regulate the expression levels of proto-oncogenes or tumor suppressors, and that many types of RBPs can participate in cancer development [7]. Aberrant expression of these RBPs can affect every step of tumorigenesis, including proliferation, apoptosis, angiogenesis, senescence, and EMT/invasion/metastasis (Figure 2).

### 3.1. Proliferation

Excessive and unregulated cell proliferation can lead to the development of a tumor cell population that gradually becomes malignant. Therefore, it is very important that a potential therapy be able to restrict and inhibit the uncontrolled growth of cancer cells. Recent research has established that RBPs can be regarded as potential drivers of cancer progression, and most of the relevant RBPs modulate tumor cell proliferation [134].

IGF2BP1 and 3 have been considered as oncogenes due to their targeting of some cancer-related mRNAs [112]. IGF2BP1 promotes proliferation via regulating their mRNA targets, such as *c-Myc*, *Ki67* and *β-TrCP1* [105,135]. IGF2BP3 also regulates target mRNAs, including cell cycle regulators (*TRIM25*, *Cyclin D1*, *D3*, and *G1*), *eIF4E-BP2*, and *Myc*; these targets are downregulated by miRNAs or ribonucleases, which bind to their 3′-UTRs, leading to accelerated cell proliferation [136,137,138,139].

CPEB1 regulates translation by shortening the 3′-UTR of a target mRNA, and these changes in translation affect cell proliferation [140]. CPEB4 is overexpressed in pancreatic ductal adenocarcinoma and melanoma [72,73]. The cancer-related upregulation of CPEB4 can affect the expression levels of its target mRNAs, which include *tPA*, *MITF*, and *RAB27A*, thereby altering tumor growth [72,73].

hnRNP A1, hnRNP A2, and hnRNP I (also known as PTBP1) are highly expressed in glioblastoma and regulate the alternative splicing of *PKM* to promote tumor cell proliferation [48,49]. hnRNP I is upregulated in liver cancer and increases tumor growth through a pathway that requires *miR-194*/hnRNP I/*Cyclin D3* signal transduction [141]. hnRNP D, also known as AUF1, binds AREs within the 3′-UTR of the *Myc* mRNA to compete with TIAR-1 and affect cell proliferation [142]. hnRNP F is upregulated in bladder cancer, where it interacts with the TPX2 protein to alter the expression levels of cell cycle-related genes, such as *Cyclin D1* and *p21*, and thereby affect cell proliferation [143].

HuR contributes to tumor proliferation by modulating mRNAs of cell cycle-related genes, such as those for *Cyclin A*, *Cyclin B1*, and *Cyclin E1* [144,145].

The La-related protein (also known as LARP3) activates the IRES of the *Cyclin D1* mRNA to increase the Cyclin D1 protein level, and this promotes cell proliferation [146]. LARP1 is ubiquitously expressed and modulates mTORC1-regulated proliferation by effectively regulating the translation of mRNAs having a 5′ terminal oligopyrimidine (TOP) [147].

RBM3, 5, 6, and 10 are generally mutated in various types of cancer, and are regarded as putative regulators of tumor progression [62,148]. These four RBMs can promote cell growth in liver and lung cancer by interacting with *SCD-circRNA* [62] or modulating alternative splicing of their target mRNAs, such as the Notch pathway regulator, *NUMB* [148].

LIN28A and LIN28B, which negatively regulate members of the *let-7* miRNA family, promote cell proliferation in cancers derived from various origins. LIN28A and LIN28B regulate proliferation by directly or indirectly binding to and affecting the expression levels of cancer growth-related genes, including *HER2* and *HMGA1*, in both *let-7*-dependent and -independent manners in breast cancer [149].

SAM68 binds intron 4 of *Cyclin D1* and triggers its alternative splicing by interfering with the binding of splicing-related factors. The alternatively spliced *Cyclin D1b* variant has higher oncogenic potential than *Cyclin D1a* [65]. The binding affinity of SAM68 to a target mRNA is enhanced by its acetylation, which occurs through inhibition of the deacetylase, HDAC, or activation of the acetyltransferase, CBP, and plays a role in tumor proliferation [150].

QKI exhibits little or no expression in colon cancers due to irregular hypermethylation. Induction of QKI expression promotes the accumulation of p27 and membrane localization of β-catenin, leading to decreases in proliferation and tumorigenesis [151].

MSI1 downregulation reduces cell proliferation by triggering cell cycle arrest at G0/G1 phase. MSI1 also binds to the 3′-UTRs of *p21* and *p27*, and reduces their expression [126]. In addition, loss of MSI1/2 affects the growth of pancreatic cancer from patient-derived tumors in mouse, reducing the expression of proto-oncogenes such as *c-Met*, *Fos*, and *Fyn* [127].

### 3.2. Apoptosis

Cancer cells not only have the ability to continually divide and grow; they are also able to avoid cell death. Apoptosis is an essential process by which normal cells decide to die under a harmful condition. However, cancer cells ubiquitously escape apoptosis to promote tumor progression. Various RBPs participate in this escape by regulating target apoptosis-related mRNAs, such as those for *p53*, *Bcl*, *Fas*, *PARP*, *Caspases*, and so on [152].

HuR regulates the stabilities of the mRNAs for *MSI1*, *SIRT1*, *Bcl-2*, *Mcl-1*, and *ProTα* to affect apoptosis [121,153,154]. HuR modulates the expression of PDCD4 through a competitive interaction with the RBP, T cell intracellular antigen-1 (TIA-1) [155]. TIA-1 promotes apoptosis by enhancing the alternative splicing of the *Fas* mRNA or stabilizing *PDCD4* [155,156]. eIF4E is involved in regulating the expression levels of *Bcl-2* and *Bcl-xL*, and thereby affects cell survival [157]. LARP family proteins contribute to suppressing cell growth by promoting apoptosis through regulation of *Bax*, *Bcl-2*, *Bik*, *Mdm2*, and *XIAP* [158,159]. CELF1 is upregulated in oral squamous cancer cell (OSCC), and its depletion in OSCC cells induced apoptosis by regulating the pro-apoptotic genes *Bad*, *Bax*, and *JunD* [160]. Enhanced expression of dead-end 1 (DND1) is detected in breast cancer cells and is correlated with prolonged survival in patients. Mechanistically, DND1 promotes apoptosis by enhancing the mRNA expression of *BIM* via a competitive interaction with *miR-221* [161]. Reduction of MSI1 diminishes cancer cell proliferation and activates Caspase 3-mediated apoptosis in colon adenocarcinoma xenografted mouse models [162]. In breast cancer, KIN17 is associated with apoptosis. Depletion of KIN17 was associated with reduced cell proliferation and increased activity of Caspase 3/7, which promotes apoptosis [163]. The RBM family member, RBM10, is associated with apoptosis through its ability to regulate the levels of p53 and cleaved PARP [164].

### 3.3. Angiogenesis

Angiogenesis is the physiological process through which new blood vessels and capillaries are formed from preexisted vessels. Cancer proliferation, survival, and metastasis require oxygen and nutrients; thus, cancer needs access to blood vessels. Early observations revealed that rapidly developing tumors are highly vascularized compared to dormant tumors, suggesting that angiogenesis is required for cancer progression. Angiogenesis is promoted by angiogenic activators, such as V*EGF*, FGF2, and TNF-α [165]. RBPs can modulate the expression of angiogenic factors to play important roles in cancer progression. 

HuR expression correlates with the grade of brain tumor [166]. High-level HuR expression is observed in rapidly growing and high-grade brain tumors, such as glioblastoma and medulloblastoma, whereas only weak HuR expression is detected in low-grade brain tumors. In perinecrotic regions of glioblastoma, significant HuR expression is detected and is linked to the expression of angiogenic growth factors. HuR has high binding affinities for the 3′-UTRs of angiogenic factors (*VEGF*, *COX-2*, *IL-8*), an immunomodulating factor (*IL-6*), *TGF-β*, and *TNF-α*, and thus plays important roles in regulating tumor proliferation and angiogenesis in the central nervous system [166]. Alternative splicing of *TIA-1* in exon 5 generates a shortened TIA-1 (sTIA-1) isoform. Compared to normal tissues, sTIA-1 is elevated in colorectal cancer and is positively correlated with the tumor stage. *VEGF-A* is subject to sTIA-1-mediated alternative splicing. Depletion of sTIA-1 or overexpression of full-length TIA-1 (flTIA-1) increases the generation of the anti-angiogenic isoform, *VEGF-A165b*, leading to decreased tumor formation and vascularization in colon cancer xenografted mice, compared to those overexpressing sTIA-1 [167]. eIF4E affects angiogenesis by regulating the translation of its target mRNAs, *VEGF*, *Cyclin D1*, and *FGF2* [168,169]. TTP functions as a tumor suppressor in colon cancer. In this disease, TTP is downregulated, whereas VEGF is highly expressed. In a human colon cancer xenograft mouse model, overexpression of TTP enhances degradation of the V*EGF* mRNA, resulting in the inhibition of tumor growth and angiogenesis [170]. LARP6 expression is elevated in breast cancers; such cancers show especially aggressive phenotypes due to the upregulations of MMP9 and VEGF [171].

### 3.4. Senescence

Cellular senescence is a multi-pronged biological process that leads to permanent cell-cycle arrest. Numerous intrinsic and extrinsic triggers, such as telomere length, genetic alterations, Reactive oxygen species (ROS) production, and chemo- or radio-therapy, can induce cellular senescence in both normal and cancer cells. Senescent cells usually share common characteristics, such as activation of the p53/p21 stress response and/or RB/p16 tumor suppressor pathways, positive staining of senescence-associated β-galactosidase (SA-β-gal), and the senescence-associated secretory phenotype (SASP) [172,173]. RBPs contribute to numerous alterations of gene expression during senescence, and can modulate tumor cell senescence. 

HuR modulates cellular senescence by destabilizing the *TIN2* mRNA and reducing its translation. Depletion of HuR leads to the mitochondrial localization of TIN2, and the resulting increase in the ROS level induces cellular senescence [174].

Wig1 decreases the *p21* mRNA level through binding to the *p21* 3′-UTR stem-loop structure near the miRNA-recognition site and facilitating *p21* mRNA decay through the recruitment of Ago2 to the target site of *p21* mRNA. Knockdown of Wig1 increases p21 expression and results in premature senescence. Moreover, the mRNA expression level of *Wig1* affects *p21* mRNA level in a human lung cancer xenografted mouse model [116].

Fragile X-related protein 1 (FXR1) is upregulated in oral squamous cancer cells. Its depletion induces cellular senescence by inactivating the phosphatidylinositol 3-kinase/Akt signaling pathway and triggering the expressions of senescence-related genes, such as *p53*, *PTEN*, *p21*, and *p27*. Overexpressed FXR1 regulates the noncoding RNA, *TERC*, to avoid cellular senescence in oral squamous cancer cells [175].

AUF1 knockout mice exhibit increased cellular senescence in various normal and tumor tissues due to enhanced levels of senescence-related genes, such as *p16*, *p19*, and *p21*, and inactivated transcription of mTERT [176].

TTP controls the levels of p53 and hTERT by promoting the rapid mRNA decay of cellular ubiquitin ligase E6-associated protein (E6-AP). The TTP protein, which is downregulated in cervical cancer compared to normal cervix, functions as a tumor suppressor by inducing senescence through the regulation of p53 protein stability [177].

SRSF1 is an oncogenic splicing factor that is upregulated in various cancers. It interacts with the RPL-MDM2 complex and increases p53 protein expression and activity by blocking the proteasome-mediated degradation of p53, leading to oncogene-induced senescence [178].

### 3.5. EMT, Invasion, and Metastasis

EMT occurs in normal tissues during embryonic development, tissue regeneration, and wound healing, as well as in tumors undergoing progression and metastatic expansion. Epithelial cells can transform to a mesenchymal cell phenotype via several biological processes. When EMT occurs, cell-to-cell adhesion and cell polarity is inhibited; this facilitates invasion and metastasis in cancer. Transcriptional and post-transcriptional alterations of gene expression regulate EMT and the downstream alterations in cell morphology and function. RBPs can manage these processes by modulating the alternative splicing, stability, translation, and polyadenylation of mRNAs during EMT [179].

ESRP1 and/or ESRP2 further contribute to EMT by regulating the alternative splicing of *FGFR2*, *Exo70*, and *CD44*. Gain- and loss-of function studies showed that ESRP1 and 2 are required for the cell to modulate the expression of epithelial *FGFR2* exon IIIb or the mesenchymal *FGFR2* exon IIIc variant in various normal and cancer tissues [58]. This conversion of *FGFR2* splicing yields a less aggressive phenotype and suppresses metastasis in pancreatic cancer [180]. ESRP1 also regulates the alternative splicing of *Exo70*, a component of the exocyst complex that tethers secretory Golgi vesicles to the plasma membrane prior to fusion. The epithelial *Exo70* variant can affect the expression levels of EMT-related genes, such as those encoding Snail and ZEB2, inducing the epithelial phenotype and inhibiting tumor metastasis in breast cancers; in contrast, the mesenchymal *Exo70* variant cooperates with the Arp2/3 complex to enhance tumor invasion by inducing actin polymerization [181]. In breast cancer, reduction of ESRP1 changes the variant expression from C*D44v* to C*D44s* and inhibits metastasis to the lung. Mechanistically, the ESRP1-mediated alternative splicing of the *CD44* mRNA stimulates the cystine transporter (x-CT), which is involved in cysteine uptake and the accumulation of reductive glutathione (GSH); this decreases ROS and thereby prevents cancer cell metastasis [60]. hnRNP M can enhance breast cancer metastasis by stimulating the expression of mesenchymal specific *CD44v* via a competitive interaction with ESRP1 [52].

hnRNP family members play various roles in EMT and cancer metastasis. A loss-of-function study showed that hnRNP A2/B1 expression is specifically associated with intermediate and mesenchymal cell lines (A549 and H1703). The reduced expression level of hnRNP A2/B1 is related to an increase in *E-cadherin* and decreases in *Twist1* and *Snail1* (an inhibitor of E-cadherin), indicating that hnRNP A2/B1 may be correlated with lung metastasis [182]. hnRNP A2 activates invasion by modulating splicing of *TP53INP2* mRNA [183]. hnRNP E1 can regulate TGFβ-mediated EMT mechanisms by binding within the 5′ or 3′-UTR of its target mRNAs, such as those for *Dab2*, *ILEI*, or phosphatase of regenerating liver (*PRL*)-3, which will lead to activation or repression of these targets [130,184]. hnRNP E1 also inhibits the translation of *Inhibin βA* by binding to its target mRNA. The translational level of Inhibin βA is elevated after the knockdown of hnRNP E1, and this increased Inhibin βA expression promotes metastasis [185]. The expression of hnRNP R may also influence metastasis and tumor aggressiveness by increasing the mRNA stability of its target, *Cyclin B1* and *CENPF* [186].

KHSRP is significantly expressed in highly metastatic non-small-cell lung carcinoma cells, and its depletion decreases tumorigenic phenotypes, including growth and metastasis, both in vitro and in vivo. KHSRP interacts with hnRNP C and then stimulates the IFN-α/JAK/STAT pathway to promote lung metastasis. Sustained KHSRP expression inhibits TGFβ-mediated EMT by activating *miR192-5p* to reduce EMT-related factors. In cooperation with hnRNP A1, KHSRP can facilitate alternative splicing to produce the epithelial-specific and EMT-related variants of *ENAH*, *CD44*, and *FGFR2* [19].

CPEB1 has an inverse correlation with metastasis of breast cancer. Reduction of CPEB1 in TGFβ-mediated EMT results in metastasis via *MMP9* mRNA polyadenylation and translation [70].

eIF4E is highly upregulated in colorectal cancer patients with metastatic tissues in liver. Reduction of eIF4E in colorectal cancer cells suppresses the invasion and migration by modulating the expression of *MMPs* and *VEGF* [187].

IGF2BP1 is specifically decreased in metastatic breast cancer due to its highly methylated promoter; it affects the migration and proliferation of metastatic cells [188]. IGF2BP2 and 3, contribute to the progression of metastatic breast cancer by modulating the formation of invadopodia, which are actin-rich protrusions of metastatic tumors that act to degrade the extracellular matrix (ECM). In triple-negative breast cancer, IGF2BP2 and 3 cooperate to promote cell migration and invasion by destabilizing the *PR* mRNA through recruitment of the CNOT1 deadenylase complex [189].

The La protein can promote the translation of the *laminin B1* (*LamB1*) mRNA by binding within its IRES, and thereby contributes to invasion and metastasis in hepatocellular carcinoma [190]. LARP7 is expressed at a low level in breast cancer and interacts with the transcription elongation factors, 7 SK snRNP and P-TEFb. Depletion of LARP7 can promote the escape of P-TEFb from the 7 SK snRNP-harboring complex; the released P-TEFb can encourage transcription elongation by phosphorylating the two negative elongation factors and promoting the activity of PolII, which leads to EMT and enhances invasion and metastasis [191].

The overexpression of LIN28A and/or LIN28B is related to enhanced tumorigenesis and invasive progression in a murine model of invasive colorectal cancer, in association the colonic stem cell markers, LGR5 and PROM1 [192].

SAM68-knockout mice have uterine defects and SAM68-heterozygous mice generated by mating with MMTV-PyMT transgenic mice, in which rapid tumor growth is induced by expression of an oncogene, exhibit delayed tumorigenesis, decreased metastasis, and induction of tyrosine kinase-related signaling [193]. SAM68 also cooperates with scaffold/matrix-associated region-binding protein 1 (SMAR1); phosphorylation of SMAR1 by ERK-1/2 kinase or downregulation of SMAR1 itself lead to increased acetylation of SAM68, which in turn affects the alternative splicing of the *CD44* mRNA and thereby modulates cancer metastasis [194].

RBM47 inhibits tumor progression and metastasis by increasing the *DKK1* [195].

SRSF1 generates the Δ*Ron* variant, and enhances EMT and cell motility in breast and colon cancers [32].

## 4. RBPs as Therapeutic Targets in Cancer

As described in detail above, RBPs can affect the overall progression of various cancer types, including aspects such as cancer cell proliferation, survival, angiogenesis, metastasis, and senescence, by regulating the expression, localization, and post-translational modification of target mRNAs (Table 1). Accordingly, researchers are constantly seeking to develop drugs that can target RBPs to treat cancer patients [196]. Here, we will review a number of candidate therapeutics, including small-molecule drugs, inhibitors, therapeutic small peptides, and antisense oligonucleotides (ASOs), that are currently being developed and applied in clinical trials (Table 2).

Small-molecule drugs can target RBP functions in various human diseases, including cancer, and are already being clinically tested and reported for their anti-cancer effects. For example, eukaryotic translation initiator factor 4F (eIF4F) is an important protein complex that recruits the small ribosomal subunit (40S) to the 5′ cap of mRNAs during cap-dependent translation initiation; this complex comprises the three proteins, eIF4E, eIF4A, and eIF4G [119]. eIF4E is a 5′ cap-dependent translation initiation factor that is reportedly overexpressed in multiple cancers, where it is associated with aggressive phenotypes. Ribavirin is an antiviral guanosine analog that is able to physically imitate the 5′ -terminal m^7^G cap-structure, and thereby interacts with the 5′ cap of the *eIF4E* mRNA. This blocks the transport and translation of eIF4E-regulated oncogenes, such as *Cyclin D1*, to reduce tumorigenesis in vitro and in vivo [197]. N-7 benzyl guanosine monophosphate tryptamine phosphoramidate prodrug (4Ei-1) prevents eIF4E cap binding and triggers proteasomal degradation of eIF4E, thereby chemosensitizing breast and lung cancer to gemcitabine [198]. 4EGI-1, 4E1RCat, and 4E2RCat disrupt the cooperation of eIF4E with eIF4G and eIF4A to inhibit cap-dependent translation and promote pro-apoptotic effects in vitro and in vivo [199,200]. Ouabain, perillyl alcohol, and mTOR inhibitors (e.g., rapamycin, Torin1, and AZD8055) also interrupt the interactions of eIF4E and eIF4G in various cancer cells [201,202].

When MnK inhibitors were applied to in vitro and in vivo models of melanoma, lymphoma, colon cancer, and lung cancer, blocking the phosphorylation of eIF4E was found to inhibit cell proliferation and metastasis [203].

HuR shows aberrant expression and localization in various types of cancer. MS-444, okicenone, dehydromutactin, DHTS, and AZA-9 are nanomolar inhibitors of HuR that block its RNA-binding activities by targeting the RRM1 and RRM2 of HuR. In the presence of these molecules, HuR does not bind the AREs of target RNAs, such as *IL-2*, *IL-1β*, *TNF-α*, and *COX-2*, and *c-fos*; this reduces their HuR-mediated mRNA stabilization. These nanomolar inhibitors of HuR show anti-cancer effects in in vitro and in vivo models of colorectal cancer [209,210,211,212]. CMLD-2, another small-molecule inhibitor, inactivates HuR-mediated RNA stabilization. In non-small-cell lung carcinoma and thyroid cancer cells, CMLD-2 treatment decreases the mRNA expression of HuR or competitively binds to HuR, and thereby downregulates target mRNAs, such as *Bax*, *Bcl-xL*, and *Mad2*. CMLD-2 treatment therefore increases apoptosis and decreases tumor aggressiveness [213,214].

High-throughput analysis to identify potential inhibitors of MSI proteins was employed for cancer therapy. From among 30,000 candidate compounds, oleic acid was found to bind the RRM1 motif in MSI and inhibit its interaction with target mRNAs by promoting conformational changes. MSI can also modulate Stearoyl-CoA desaturase-1 which is required for fatty acid synthesis, eventually affecting cell division [217]. In another screening assay used to search for an inhibitor of MSI, (−)-gossypol, a natural compound known to have an anti-tumor effects in various cancers, was found to cooperate with MSI and inhibit tumor growth by enhancing apoptosis and autophagy in a mouse xenograft model [218]. Ro 08-2750 (Ro) is a novel small molecule that selectively inhibits MSI2 activity. It binds to RRM1 of MSI2 to disrupt its interaction with RNA. The Ro-mediated inactivation of MSI2 reduces the expression of target mRNAs, including those for *TGF-βR1*, *c-Myc*, *Smad3*, and *HOXA9*. In addition, Ro can inhibit leukemogenesis in both in vitro and in vivo models [219].

Overexpression of LIN28 in cancer inhibits the maturation of *let-7* to promote tumor growth. Using a protein/RNA FRET assay, researchers identified compound 1632 as an inhibitor of LIN28. Compound 1632 inhibits the interaction of LIN28 with pre-*let-7* and induces the maturation of *let-7*, resulting in disruption of tumorigenesis in vitro and in vivo in xenograft mice [221]. High-throughput screening identified a number of other LIN28 inhibitors, including compound 1, KCB3602, LI71, and TPEN. Compound 1, KCB3602, and LI71 interact with the CSD of LIN28 to block its interaction with *let-7*. TPEN destabilizes the ZKD of LIN28. These inhibitors disrupt LIN28-mediated oligouridylation to restore the level of *let-7* in cancer and embryonic stem cells [222].

Therapeutic peptides comprise 55 or fewer amino acids; compared to antibodies, they are easier to synthesize and exhibit higher cell penetration and lower immunogenicity [223]. In ovarian cancer, 4EBP-based therapeutic peptides can bind eIF4E to prevent its cap-dependent translation, thereby disrupting tumor growth. This eIF4E-targeting peptide was further merged with an analogue of gonadotropin-releasing hormone (GnRH), which is expressed in the majority of ovarian cancer patients and is known for its anti-cancer effects. The GnRH-4EBP fusion peptide was reported to inhibit tumor growth without inducing any cytotoxicity in a xenograft model of epithelial ovarian cancer [204]. Synthetic peptides have also been used to interrupt the RBM38-eIF4E interaction, with the goal of suppressing translation of the *p53* mRNA. Using this strategy, p53 levels were upregulated and tumor growth was inhibited in vitro and in vivo [205].

ASOs and small-interfering RNAs (siRNAs) are commonly utilized to regulate gene expression and have been applied to many diseases as nucleic acid drugs. Both oligonucleotides can bind their target RNAs by Watson-Crick base pairing to modulate splicing, target miRNAs, and inhibit translation [224]. Several FDA-approved or clinically tested drugs take advantage of these characteristics to engage in gene-specific silencing for the treatment of human diseases, including neurological diseases and viral infections [225]. Although no such drug has yet been approved for cancer treatment, many researchers are conducting relevant clinical and nonclinical studies, especially in the context of targeting RBPs. For example, ISIS 183750, which is an ASO drug against eIF4E, disrupts the proliferation of colorectal cancer cells and shows a synergetic effect with the chemical drug, irinotecan. Combination therapy with ISIS 183750 and irinotecan has been tested in phase I/II clinical trials among patients with advanced solid tumors and irinotecan-refractory colorectal cancer [206]. Another ASO, LY2275796, advanced to preclinical studies in mice, where it dose-dependently reduced eIF4E protein and tumors. In humans, a phase I clinical trial has been completed in patients with advanced tumors, and combination therapies with chemical drugs are currently in phase II trials [207]. siRNAs against eIF4E can inhibit tumor growth and stimulate the cytotoxic effects of cisplatin in human breast cancer in vitro and in vivo [208]. HuR can also be targeted with siRNAs, and a chemo-biologic combinatorial drug delivery system can be used to actively target the desired cells or tissues. A siRNA against HuR was loaded into a folic acid (FA)-conjugated polyamidoamine dendrimer (Den)-based nanoparticle, and this formulation was found to effectively decrease HuR expression and cell proliferation in lung cancer cells. In combination with cis-diamine platinum (CDDP), this nanoparticle exhibited synergistically improved anti-tumor effects with reduced cytotoxicity [215]. Similarly, when a siRNA against HuR was loaded to a transferrin receptor-targeted liposomal nanoparticle delivery system, HuR expression was disrupted and tumor growth was inhibited in a mouse lung cancer model [216]. Additionally, ASOs and siRNAs against MSI could inhibit the tumor growth of pancreatic and ovarian cancers in vitro and in vivo [127,220].

RBPs also have potential as a diagnostic and prognostic markers for different types of cancers due to their abnormal expression and functions for mRNA regulation. For example, IGF2BP3 is mostly upregulated and related with invasive phenotypes or poor prognosis in a variety of cancers [226,227]. In addition, cytoplasmic HuR expression is connected with more aggressive phenotypes and poor survival rates in ductal breast cancer from patients [228]. Upregulation of hnRNP C in gastric cancer induces chemoresistance, which is negatively associated with overall survival in patients [229]. Furthermore, research is ongoing to discover RBPs for using a diagnostic and prognostic markers that show cancer-specific expression using advanced bioinformatic tools such as microarray or newly analyzing RNA-seq data based on the Cancer Genome Atlas (TCGA) data [230,231].

## 5. Conclusions

Considerable evidence indicates that dysregulation of RBPs occurs in various human cancer types and affects every step of cancer development. RBPs can modulate gene expression at the post-transcriptional and translational levels to induce or reduce cancer-related genes. A number of RBPs have been found to simultaneously regulate numerous genes related to cancer development, resulting in diverse changes in cancer progression and suggesting targets for new therapeutic approaches. With the development of deep-running technologies, such as single-cell analysis and crosslinking and immunoprecipitation (CLIP), many new RBPs and their partners have been discovered and subjected to functional studies. Based on the results of these studies, researchers have attempted to target RBPs and/or their partners in clinical and non-clinical studies using siRNAs, ASOs, and small molecules. Some of these strategies have been found to prevent cancer development and growth, thereby yielding a therapeutic effect. However, there are still some limitations that must be overcome. More research should be conducted to elucidate the detailed functions of the discovered RBPs and to develop specific methods for targeting them in cancer treatment without affecting neighboring normal cells. In addition, it would be useful to improve the target specificity of drug delivery systems with the goal of further enhancing the effects of the developed drugs [232]. In conclusion, we need to develop a more comprehensive knowledge of RBPs in cancer, as they hold great promise as therapeutic targets for the treatment of cancer in the near future.

## Figures and Tables

**Table 1:** Roles of RNA binding proteins (RBPs) in cancer.

RBP	Cancer Types	Expression	Mechanisms	Targets	Cancer Traits	References
LIN28A/B	Brain, breast, colon, cervical esophageal, head and neck, liver, lung, lymphoma, renal, prostate, ovary	Upregulated	miRNA processing	*let-7a*	Proliferation, invasion, metastasis, angiogenesis	[9,10,11,12,13,14,15,149,192]
SMAD	Colon	Upregulated	miRNA processing	*miR-21*, *miR-199a*	Invasion, metastasis	[25,26]
SRSF1 (SF2/ASF)	Lung, colon, kidney, liver, pancreas, breast, prostate	Upregulated	Alternative splicing	*Ron*, *BIN1*, *MKN2*, *S6K1*, *Bcl-x*, *Mcl-1*, *CASP2*, *CASP9*, *ICAD*, *BIM*, *TEAD1*, *VEGF*, *RPL-MDM2*	Senescence, EMT, invasion, metastasis, proliferation, angiogenesis	[32,33,34,35,36,37,38,178]
SRSF3 (SRp20)	Lung, breast, stomach, skin, bladder, colon, thyroid, kidney, brain	Upregulated	Alternative splicing	*FoxM2*, *HIPK2*, *ETV1*, *NDE1*, *HNF1a*, *ERN1*, *HMGCS1*, *DHCR7*, *SCAP*, *LIFR*, *Epb4.1l5*, *Myo1b*, *CTNND1*, *GIT2*, *SLK*, *SP4*	Proliferation, apoptosis, EMT, metastasis	[39,40,41,42,43,44]
SRSF6 (SRp55)	Lung, colon	Upregulated	Alternative splicing	*INSR*, *DLG1*, *MKNK2*	Proliferation	[45]
SPF45 (RBM17)	Leukemia, cervical	Upregulated	Alternative splicing	*Fas*	Apoptosis	[46]
RBM3	Liver	Upregulated	circRNA biogenesis	*SCD-circRNA*	Proliferation	[61,62]
RBM5, 6, 10	Liver, lung	Upregulated or downregulated	Alternative splicing	*NUMB*, *p53*, *C-PARP*	Proliferation, apoptosis	[148,164]
RBM47	Breast, lung	Downregulated	mRNA stability	*DKK1*	Metastasis	[195]
KHSRP	Brain, breast, esophageal, liver, lung, renal, testis, thyroid	Upregulated or downregulated	miRNA processing	*miR-26a/b*, *let-7a*, *miR-23a*, *miR-192-5p*, *miR-21*, *miR-130b*, *miR-301*	EMT, invasion, metastasis	[16,17,18,19,20,21]
hnRNPA1	Brain, breast, colon, liver, lung, pancreas, cervical, gastric	Upregulated	miRNA processing	*miR-18a*, *let-7a*	Proliferation	[19,22,24,47,48,49,50]
hnRNPA2/B1	Brain, lung	Upregulated	Alternative splicing	*BIN1*, *WWOX*, *CFLAR*, *CASP9*, *CD44*, *TP53IP2*, *E-cadherin*, *Twist*, *Snail1*	Proliferation, EMT, metastasis	[47,48,49,51,182,183]
hnRNP D (AUF1)	Breast, colon, gastric, liver, lung, pancreas, renal, sarcoma, thyroid	Upregulated	mRNA stability	*Cyclin D1*, *p21*, *p16*, *Bax*, *Bcl-2*, *Gadd45a*, *CASP2*, *MMP9*, *FGF9*, *Fos*, *TYMS*, *JunD*, *Myc*, *lncRNA NEAT1*	Proliferation, Senescence	[86,87,88,89,90,91,92,142,176]
hnRNP E1/2 (PCBP1/2)	Breast, lung, colon, pancreas	Upregulated or downregulated	mRNA stability, translation	*Dab2*, *ILEI*, *PRL-3*, *Inhibin βA*, *p73*, *GLS2*	Senescence, EMT, invasion, metastasis	[129,130,184,185]
hnRNP F	Bladder	Upregulated	mRNA stability	*TPX2*	Proliferation	[143]
hnRNP M	Breast	Upregulated	Alternative splicing	*CD44*	EMT, invasion, metastasis	[52]
hnRNP H	Brain	Upregulated	Alternative splicing	*Ron*, *MST1R*	Invasion, migration	[53]
hnRNP I (PTB)	Breast, ovarian, brain, colon	Upregulated	Alternative splicing	*FGFR-1*, *USP5*, *PKM*, *Cyclin D3*	Proliferation	[48,49,55,56,57,122,141]
hnRNP K	Breast	Upregulated	Subcellular localization	*lncRNA MALAT1*	Metastasis	[82,83]
hnRNP R	Gastric	Upregulated	mRNA stability	*Cyclin B1*, *CENPF*	EMT, invasion, metastasis	[186]
CELF1	Oral	Upregulated	mRNA stability	*Bad*, *Bax*, *JunD*	Apoptosis	[160]
DND1	Breast	Downregulated	mRNA stability	*BIM*	Apoptosis	[161]
FXR1	Oral	Upregulated	mRNA stability	*PI3K/AKT*, *p53*, *PTEN*, *p21*, *p27*, *TERC*	Senescence	[175]
ESRP 1/2	Breast, colon, head and neck, lung, pancreas, renal, melanoma	Upregulated	Alternative splicing	*FGFR2*, *CD44*, *CTNND1*, *ENAH*, *Snail*	EMT, invasion, metastasis	[52,58,59,60,180,181]
SAM68	Prostate, renal, breast, colon, cervical	Upregulated	Alternative splicing	*CD44*, *Cyclin D1*, *Bcl-x*	Proliferation, EMT, invasion, metastasis	[63,64,65,66,150,193,194]
CPEB1	Breast, liver	Upregulated	Alternative polyadenylation	*MMP9*, *VEGF*, *HIF-1α*	Proliferation, angiogenesis, EMT, invasion, metastasis	[70,71,74,77,140]
			Subcellular localization	*ZO-1*	EMT	
CPEB2	Breast	Upregulated	Alternative polyadenylation	*HIF-1α*	Metastasis	[71]
CPEB4	Breast, liver, melanoma, pancreas	Upregulated	Alternative polyadenylation	*MIFT*, *RAB7A*, *VEGF*, *tPA*	Proliferation, angiogenesis	[72,73,74]
IGF2BP1 (IMP1/ZBP1)	Breast, colon, lung, melanoma, ovary, skin, liver	Upregulated	Subcellular localization	*β-actin*, *E-cadherin*, *α-actinin*, *Arp-16*	Proliferation, EMT, invasion, metastasis	[78,79,105,106,107,108,111,112,135,188]
			mRNA stability	*β-TrCP1*, *CD44*, *lncRNA HULC*		
IGFBP2 (IMP2)	Brain, breast, leukemia, lung, colon	Upregulated	Subcellular localization	*NDUFS3*, *COX7b*	EMT, invasion, metastasis	[80,109,112,189]
			mRNA stability	*RAF1*, *PR*		
IGF2BP3 (IMP3)	Breast, colon, leukemia, lung, ovary, pancreas, renal, liver	Upregulated	mRNA stability	*HMGA2*, *LIN28*, *Myc*, *PR*	Proliferation, EMT, invasion, metastasis	[110,112,136,137,138,139,189]
HuR	Brain, breast, cervical, colon, gastric, liver, leukemia, prostate, ovary, gallbladder	Upregulated in most cancers	mRNA stability	*Cyclin (A*, *B1*, *D1*, *and E)*, *IL-1β*, *IL-2*, *TNF-α*, *IL-8*, *MMP9*, *HIF-1α*, *VEGF*, *SIRT1*, *Snail*, *c-Myc*, *Wnt5a*, *COX-2*, *c-fos*, *p21*, *p16*, *p27*, *TIN2*, *MSI1*, *Bcl-2*, *Bcl-xL*, *Mcl-1*, *ProTα* , *lncRNA NEAT1*, *lncRNA-HGBC*, *OIP5-AS1*, *LincRNA-p21*, *HOTAIR*	Proliferation, apoptosis, angiogenesis, senescence, invasion, metastasis	[93,94,95,96,97,98,99,120,121,122,123,128,144,145,153,154,155,166,174]
			mRNA translation	*ProTα*, *p53*, *MSI1*, *HIF-1α*, *XIAP*		
eIF4E	B-cell Lymphoma, breast, colon, lymphoma, melanoma	Upregulated	Translation	*Bcl-2*, *Bcl-xL*, *VEGF*, *FGF2*, *MMP2*, *MMP9*	Apoptosis, angiogenesis, EMT, invasion, metastasis	[119,157,168,169,187]
KIN17	Breast, cervical	Upregulated	mRNA translation	*Caspase 3/7*	Apoptosis	[163]
La	Cervical, liver	Upregulated	Translation	*Cyclin D1*, *LamB1*	Proliferation	[146,190]
LARP 1	Cervical, lung	Upregulated	mRNA stability, translation	*Bax*, *Bcl-2*, *Bik*, *Mdm2*, *XIAP*, *5*′ *TOP mRNAs*	Proliferation, apoptosis	[147,158,159]
LARP6	Breast	Upregulated	Translation	*MMP9*, *VEGF*	Angiogenesis	[171]
LARP 7	Breast	Downregulated	Stability	*P-TEFb*, *7 SK snRNP*	EMT, invasion, metastasis	[191]
MSI 1/2	Bone, Liver	Upregulated	mRNA translation	*BRD4*, *c-Met*, *HMGA2*, *NUMB*, *Fos*, *Fyn*, *p21*, *p27*, *Caspase 3*	Proliferation, apoptosis, EMT, invasion, metastasis	[124,125,126,127,162]
QKI	Breast, colon	Upregulated	circRNA biogenesis	*SMARCAS5*, *POLE2*, *OXNAD1*, *SHPRH*, *SMAD2*, *ATXN2*, *DOCK1*, *GNB1*	Invasion, metastasis	[67]
		Downregulated	mRNA translation	*p27*, *β-catenin*	Proliferation	[151]
PDCD4	Leukemia, breast, colon	Downregulated	mRNA translation	*A-Myb*	Proliferation	[131]
TIA-1	Breast, colon, cervical	Upregulated	Alternative splicing, mRNA stability, Translation	*Fas*, *VEGF*, *PDCD4*, *GADD45A*	Apoptosis, angiogenesis	[155,156,167]
TTP	Brain, breast, colon, leukemia, liver, melanoma, pancreas, prostate	Upregulated or downregulated	mRNA stability	*Cyclin B1*, *Cyclin D1*, *Bcl-2*, *cIAP*, *VEGF*, *Snail*, *Twist1*, *ZEB1*, *SOX9*, *MACC1*, *MMP2*, *MMP9*, *IL-6*, *PD-L1*	Angiogenesis, senescence	[100,101,102,103,104,170,177]
Wig1 (ZMAT3)	Breast, lung, osteosarcoma	Downregulated	mRNA stability	*p53*, *p21*, *ACOT7*	Senescence	[113,114,115,116,117]
			Translation	*ACOT7*		

**Table 2:** Therapeutic strategies of RNA binding proteins (RBPs) in cancer.

RBP	Therapeutic Types	Compounds	Functions	Cancer Types	References
eIF4E	Small molecule inhibitor	Ribavirin	Mimic of 5′ 7-methyl guanosine cap-structure. Interacts with eIF4E and inhibits eIF4E-mediated translation of oncogenes	Human squamous cell carcinoma (HSCC), acute myeloid leukemia (AML)	[197]
		4Ei-1	Converts to 7Bn-GMP. Antagonizes eIF4E cap binding and induces eIF4E proteasomal degradation, inhibiting translation initiation	Breast, lung, mesothelioma	[198]
		4EGI-1, 4E1RCat, 4E2RCat, Ouabain, Perillyl alcohol, Rapamycin, Torin1, AZD8055	Interferes with association of eIF4E and eIF4G, inhibiting translation initiation	Lung, melanoma, lymphoma, colon, liver, breast, prostate	[199,200,201,202]
		MnK inhibitors	Inhibits phosphorylation of eIF4E	Melanoma, lymphoma, colon, lung	[203]
	Therapeutic peptides	GnRH-4EBP	Binds to eIF4E and disrupts eIF4E interacting with eIF4G	Ovary	[204,205]
		Pep8	Interrupts RBM38-eIF4E and increases p53 translation	Colon, breast	
	ASO	ISIS 183750	Synergetic effect with chemical drugs, such as irinotecan	Colon	[206,207]
		LY2275796	Combination therapy with chemical drugs or radiation	Prostate, lung	
	siRNA		Stimulates the cytotoxic effects of cisplatin	Breast	[208]
HuR	Small molecule inhibitor	MS-444, okicenone, dehydromutactin, DHTS, AZA-9	Targets RRM1 and 2 of HuR, and inhibits RNA-binding activities of HuR	Pancreas, colon, melanoma, brain, breast	[209,210,211,212]
		CMLD-2	Downregulates the expression of HuR Competitively interacts with HuR and inhibits its interaction with target mRNAs	Lung, thyroid	[213,214]
	siRNA		Enhances the efficiency with chemo-biologic combinatorial drug delivery system	Lung	[215,216]
MSI	Small molecule inhibitor	Oleic acid	Binds to RRM1 of MSI and induces conformational change of MSI1, inhibiting the interaction between MSI and target RNAs	Brain (CNS)	[217]
		(-)-gossypol	Interacts with RRM1 of MSI1 and block MSI1 RNA binding	Colon	[218]
		Ro 80-2750 (Ro)	Selectively binds to RRM1 of MSI2 and disrupts MSI2 interacting with RNA	AML	[219]
	ASO		Targets MSI with undruggable approaches	Ovary, pancreas	[127,220]
	siRNA		Targets MSI with liposomal preparation	Colon	[127,220]
LIN28	Small molecule inhibitor	Compound 1632	Inhibits the interaction of LIN28/pre-*let-7* and induces the maturation of *let-7*	Prostate, liver	[221]
		Compound 1, KCB3602, LI71	Binds to cold shock domain (CSD) of LIN28 and inactivates LIN28 binding against *let-7*	Ovary	[222]
		TPEN	Binds to and destabilize zinc-knuckle domain (ZKD) of LIN28. TPEN binding disrupts LIN28-mediated oligouridylation and elevates the level of *let-7*	Ovary, placenta	[222]

## References

[1] Gerstberger S., Hafner M., Tuschl T. (2014). A census of human RNA-binding proteins. Nat. Rev. Genet..

[2] Hentze M.W., Castello A., Schwarzl T., Preiss T. (2018). A brave new world of RNA-binding proteins. Nat. Rev. Mol. Cell Biol..

[3] Hong S. (2017). RNA binding proteins as an emerging therapeutic target for cancer prevention and treatment. J. Cancer Prev..

[4] Lunde B.M., Moore C., Varani G. (2007). RNA-binding proteins: Modular design for efficient function. Nat. Rev. Mol. Cell Biol..

[5] Moore S., Järvelin A.I., Davis I., Bond G.L., Castello A. (2018). Expanding horizons: New roles for non-canonical RNA-binding proteins in cancer. Curr. Opin. Genet. Dev..

[6] Lukong K.E., Chang K., Khandjian E.W., Richard S. (2008). RNA-binding proteins in human genetic disease. Trends Genet..

[7] Pereira B., Billaud M., Almeida R. (2017). RNA-binding proteins in cancer: Old players and new actors. Trends Cancer.

[8] Van Kouwenhove M., Kedde M., Agami R. (2011). MicroRNA regulation by RNA-binding proteins and its implications for cancer. Nat. Rev. Cancer.

[9] Loughlin F.E., Gebert L.F.R., Towbin H., Brunschweiger A., Hall J., Allain F.H. (2011). Structural basis of pre-let-7 miRNA recognition by the zinc knuckles of pluripotency factor Lin28. Nat. Stuct. Mol. Biol..

[10] Piskounoa E., Viswanathan S.R., Janas M., LaPierre R.J., Daley G.Q., Sliz P., Gregory R.I. (2008). Determinants of microRNA processing inhibition by the developmentally regulated RNA-binding protein Lin28. J. Biol. Chem..

[11] Heo I., Joo C., Cho J., Ha M., Han J., Kim V.N. (2008). Lin28 mediates the terminal uridylation of let-7 precursor microRNA. Mol. Cell.

[12] Heo I., Joo C., Kim Y.K., Ha M., Yoon M.J., Cho J., Yeom K.H., Han J., Kim V.N. (2009). TUT4 in concert with Lin28 suppresses microRNA biogenesis through pre-microRNA uridylation. Cell.

[13] Ustianenko D., Hrossova D., Potesil D., Chalupnikova K., Hrazdilova K., Pachernik J., Cetkobska K., Uldrijan S., Zdrahal Z., Vanacova S. (2013). Mammalian DIS3L2 exoribonuclease targets the uridylated precursors of let-7 miRNAs. RNA.

[14] Viswananthan S.R., Powers J.T., Einhorn W., Hoshida Y., Ng T.L., Toffanin S., O’Sullivan M., Lu J., Phillips L.A., Lockhart V.L. (2009). Lin28 promotes transformation and is associated with advanced human malignancies. Nat. Genet..

[15] Balzeau Z., Menezes M.R., Cao S., Hagan J.P. (2017). The LIN28/let-7 pathway in cancer. Front. Genet..

[16] Trabucchi M., Briata P., Garcia-Mayoral M., Haase A.D., Filipowicz W., Ramos A., Gherzi R., Rosenfeld M.G. (2009). The RNA-binding protein KSRP promotes the biogenesis of a subset of microRNAs. Nature.

[17] Tong L., Luo Y., Wei T., Guo L., Wang H., Zhu W., Zhang J. (2016). KH-type splicing regulatory protein (KHSRP) contributes to tumorigenesis by promoting miR-26a maturation in small cell lung cancer. Mol. Cell Biochem..

[18] Chien M.H., Lee W.J., Yang Y.C., Li Y.L., Chen B.R., Cheng T.Y., Yang P.W., Wang M.Y., Jan Y.H., Lin Y.K. (2017). KSRP suppresses cell invasion and metastasis through miR-23a-mediated EGR3 mRNA degradation in non-small cell lung cancer. Biochim. Biophys. Acta Gene Regul. Mech..

[19] Puppo M., Bucci G., Rossi M., Giovarelli M., Bordo D., Moshiri A., Gorlero F., Gherzi R., Briata P. (2016). miRNA-mediated KHSRP silencing rewires distinct post-transcriptional programs during TGF-β-induce epithelial-to-mesenchymal transition. Cell Rep..

[20] Dhamija S., Keuhne N., Winzen R., Doerrie A., Dittrich-Breiholz O., Thakur B.K., Holtmann H. (2011). Interleukin-1 activates synthesis of interleuin-6 by interfering with a KH-type splicing regulatory protein (KSRP)-dependent translational silencing mechanism. J. Biol. Chem..

[21] Fujita Y., Masuda K., Hamada J., Shoda K., Naruto T., Hmamda S., Miyakami Y., Kohmoto T., Watanabe M., Takahashi R. (2017). KH-type splicing regulatory protein is involved in esophageal squamous cell carcinoma progression. Oncotarget.

[22] Kooshapur H., Choudhury N.R., Simon B., Mühlbauer M., Jussupow A., Fernandez N., Jones A.N., Dallmann A., Gabel F., Camilloni C. (2018). Structural basis for terminal loop recognition and stimulation of pri-miRNA-18a processing by hnRNP A1. Nat. Commun..

[23] Shen K., Cao Z., Zhu R., You L., Zhang T. (2019). The dual functional role of MicroRNA-18a (miR-18a) in cancer development. Clin. Transl. Med..

[24] Michlewski G., Cáceres J.F. (2010). Antagonistic role of hnRNP A1 and KSRP in the regulation of let-7a biogenesis. Nat. Struct. Mol. Biol..

[25] Davis B.N., Hiyard A.C., Nguyen P.H., Lagna G., Hata A. (2010). Smad proteins bind a conserved RNA sequence to promote microRNA maturation by Drosha. Mol. Cell.

[26] Asangani I.A., Rasheed S.A.K., Nikolova D.A., Leupold J.H., Colburn N.H., Post H., Allgayer H. (2018). MicroRNA-21 (miR-21) post-transcriptionally downregulates tumor suppressor Pdcd4 and stimulates invasion, intravasation and metastasis in colorectal cancer. Oncogene.

[27] Nilsen T.W., Graveley B.R. (2010). Expansion of the eukaryotic proteome by alternative splicing. Nature.

[28] Biamonti G., Catillo M., Pignataro D., Montecucco A., Ghigna C. (2014). The alternative splicing side of cancer. Semin. Cell Dev. Biol..

[29] Dvinge H., Kim E., Abdel-Wahab O., Bradley R.K. (2016). RNA splicing factors as oncoproteins and tumor suppressors. Nat. Rev. Cancer.

[30] Long J.C., Caceres J.F. (2009). The SR protein family of splicing factors: Master regulators of gene expression. Biochem. J..

[31] Black D.L. (2003). Mechanisms of alternative pre-messenger RNA splicing. Annu. Rev. Biochem..

[32] Ghigna C., Giordano S., Shen H., Benvenuto F., Castiglioni F., Comoglio P.M., Green M.R., Riva S., Biamonti G. (2005). Cell motility is controlled by SF2/ASF through alternative splicing of the Ron protooncogene. Mol. Cell.

[33] Karni R., Stanchina E.D., Lowe S.W., Sinha R., Mu D., Krainer A.R. (2007). The gene encoding the splicing factor SF2/ASF is a protooncogene. Nat. Struct. Mol. Biol..

[34] Moore M.J., Wang Q., Kennedy C.J., Silver P.A. (2010). An alternative splicing network links cell-cycle control to apoptosis. Cell.

[35] Li X., Wang J., Manley J.L. (2005). Loss of splicing factor ASF/SF2 induces G2 cell cycle arrest and apoptosis, but inhibits internucleosomal DNA fragmentation. Genes Dev..

[36] Tripathi V., Ellis J.D., Shen Z., Song D.Y., Pan Q., Watt A.T., Freier S.M., Bennett C.F., Sharma A., Bubulya P.A. (2010). The nuclear-retained noncoding RNA MALAT1 regulates alternative splicing by modulating SR splicing factor phosphorylation. Mol. Cell.

[37] Malakar P., Shilo A., Mogilevsky A., Stein I., Pikarsky E., Nevo Y., Benyamini H., Elgavish S., Zong X., Prasanth K.V. (2017). Long noncoding MALAT1 promotes hepatocellular carcinoma development by SRSF1 upregulation and mTOR activation. Cancer Res..

[38] Barbagallo D., Caponnetto A., Brex D., Mirabella F., Barbagallo C., Lauretta G., Morrone A., Certo F., Broggi G., Caltabiano R. (2019). CircSMARCA5 regulates VEGFA mRNA splicing and angiogenesis in glioblastoma multiforme through the binding of SRSF1. Cancers.

[39] Jia R., Li C., McCoy J.P., Deng C.X., Zheng Z.M. (2010). SRp20 is a proto-oncogene critical for cell proliferation and tumor induction and maintenance. Int. J. Biol. Sci..

[40] Kurokawa K., Akaike Y., Masuda K., Kuwano Y., Nishida K., Yamagishi N., Kajita K., Tanahashi T., Rokutan K. (2014). Downregulation of serine/arginine-rich splicing factor 3 induces G1 cell cycle arrest and apoptosis in colon cancer cells. Oncogene.

[41] Song X., Wan X., Huang T., Zeng C., Sastry N., Wu B., James C.D., Horbinski C., Nakano I., Zhang W. (2019). SRSF3-regualed RNA alternative splicing promotes glioblastoma tumorigenicity by affecting multiple cellular processes. Cancer Res..

[42] Sen S., Jumaa H., Webster N.J.G. (2013). Splicing factor SRSF3 is crucial for hepatocyte differentiation and metabolic function. Nat. Commun..

[43] Sen S., Langiewicz M., Jumaa H., Webster N.J.G. (2015). Depletion of serine/arginine-rich splicing factor 3 in hepatocytes predisposes to hepatocellular carcinoma in mice. Hepatology.

[44] Meng N., Chen M., Chen D., Chen X.H., Wang J.Z., Zhu S., He Y.T., Zhang X.L., Lu R.X., Yan G.R. (2020). Small protein hidden in lncRNA *LOC90024* promotes “Cancerous” RNA splicing and tumorigenesis. Adv. Sci..

[45] Cohen-Eliav M., Golan-Gerstl R., Siegfried Z., Anderson C.L., Thorsen K., Ørntoft T.F., Mu D., Karni R. (2013). The splicing factor SRSF6 is amplified and is an oncoprotein in lung and colon cancers. J. Pathol..

[46] Villamizar O., Chambers C.B., Riberdy J.M., Persons D.A., Wilber A. (2016). Long noncoding RNA Saf and splicing factor 45 increase soluble Fas and resistance to apoptosis. Oncotarget.

[47] Chaudhury A., Chander P., Howe P.H. (2010). Heterogeneous nuclear ribonucleoproteins (hnRNPs) in cellular processes: Focus on hnRNP E1’s multifunctional regulatory roles. RNA.

[48] Clower C.V., Chatterjee D., Wang Z., Cantley L.C., Heiden M.G.V., Krainer A.R. (2010). The alternative splicing repressors hnRNP A1/A2 and PTB influence pyruvate kinase isoform expression and metabolism. Proc. Natl. Acad. Sci. USA.

[49] David C.J., Chen M., Assanah M., Canoll P., Manley J.L. (2010). HnRNP proteins controlled by c-Myc deregulate pyruvate kinase mRNA splicing in cancer. Nature.

[50] Zhou Z.J., Dai Z., Zhou S.L., Fu X.T., Zhao Y.M., Shi Y.H., Zhou J., Fan J. (2013). Overexpression of hnRNP A1 promotes tumor invasion through regulating CD44v6 and indicates poor prognosis for hepatocellular carcinoma. Int. J. Cancer.

[51] Golan-Gerstl R., Cohen M., Shilo A., Suh S.S., Bakàcs A., Coppola L., Karni R. (2011). Splicing factor hnRNP A2/B1 regulates tumor suppressor gene splicing and is an oncogenic driver in glioblastoma. Cancer Res..

[52] Xu Y., Gao X.D., Lee J.H., Huang H., Tan H., Ahn J., Reinke L.M., Peter M.E., Feng Y., Gius D. (2014). Cell type-restricted activity of hnRNPM promotes breast cancer metastasis via regulating alternative splicing. Genes Dev..

[53] Lefave C.V., Squatrito M., Vorlova S., Rocco G.L., Brennan C.W., Holland E.C., Pan Y.X., Cartegni L. (2011). Splicing factor hnRNPH drives an oncogenic splicing switch in gliomas. EMBO J..

[54] Xue Y., Zhou Y., Wu T., Zhu T., Ji X., Kwon Y.S., Zhang C., Yeo G., Black D.L., Sun H. (2010). Genome-wide analysis of PTB-RNA interactions reveals a strategy used by the general splicing repressor to modulate exon inclusion or skipping. Mol. Cell.

[55] Jin W., McCutcheon I.E., Fuller M., Huang E.S., Cote G.J. (2000). Fibroblast growth factor receptor-1 alpha-exon exclusion and polypyrimidine tract-binding protein in glioblastoma multiforme tumors. Cancer Res..

[56] Yamaguchi F., Saya H., Bruner J.M., Morrison R.S. (1994). Differential expression of two fibroblast growth factor-receptor genes Is associated with malignant progression in human astrocytomas. Proc. Natl. Acad. Sci. USA.

[57] Izaguirre D.I., Zhu W., Hai T., Cheung H.C., Krahe R., Cote G.J. (2012). PTBP1-dependent regulation of USP5 alternative RNA splicing plays a role in glioblastoma tumorigenesis. Mol. Carcinog..

[58] Warzecha C.C., Sato T.K., Nabet B., Hogenesch J.B., Carstens R.P. (2009). ESRP1 and ESRP2 are epithelial cell-type-specific regulators of FGFR2 splicing. Mol. Cell.

[59] Warzecha C.C., Shen S., Xing Y., Carstens R.P. (2009). The epithelial splicing factors ESRP1 and ESRP2 positively and negatively regulate diverse types of alternative splicing events. RNA Biol..

[60] Yae T., Tsuchihashi K., Ishimoto T., Motohara T., Yoshikawa M., Yoshida G.J., Wada T., Masuko T., Mogushi K., Tanaka H. (2012). Alternative splicing of CD44 mRNA by ESRP1 enhances lung colonization of metastatic cancer cell. Nat. Commun..

[61] García-Cárdenas J.M., Guerrero S., López-Cortés A., Armendáriz-Castillo I., Guevara-Ramírez P., Pérez-Villa A., Yumiceba V., Zambrano A.K., Leone P.E., Paz-y-Miño C. (2019). Post-transcriptional regulation of colorectal cancer: A focus on RNA-binding proteins. Front. Mol. Biosci..

[62] Dong W., Dai Z.H., Liu F.C., Guo X.G., Ge C.M., Ding J., Liu H., Yang F. (2019). The RNA-binding protein RBM3 promotes cell proliferation in hepatocellular carcinoma by regulating circular RNA SCD-circRNA 2 production. EBioMedicine.

[63] Bielli P., Busà R., Paronetto M.P., Sette C. (2011). The RNA-binding protein Sam68 is a multifunctional player in human cancer. Endocr. Relat. Cancer.

[64] Matter N., Herrlich P., König H. (2002). Signal-dependent regulation of splicing via phosphorylation of Sam68. Nature.

[65] Paronetto M.P., Cappellari M., Busà R., Pedrotti S., Vitali R., Comstock C., Hyslop T., Knudsen K.E., Sette C. (2010). Alternative splicing of the cyclin D1 proto-oncogene is regulated by the RNA-binding protein Sam68. Cancer Res..

[66] Paronetto M.P., Achsel T., Massiello A., Chalfant C.E., Sette C. (2007). The RNA-binding protein Sam68 modulates the alternative splicing of Bcl-x. J. Cell Biol..

[67] Conn S.J., Pillman K.A., Toubia J., Conn V.M., Salmanidis M., Phillips C.A., Roslan S., Screiber A.W., Gregory P.A., Goodall G.J. (2015). The RNA binding protein quaking regulates formation of circRNA. Cell.

[68] Erson-Bensan A.E.E., Can T. (2016). Alternative polyadenylation: Another foe in cancer. Mol. Cancer Res..

[69] Fernández-Miranda G., Méndez R. (2012). The CPEB-family of proteins, translational control in senescence and cancer. Ageing Res. Rev..

[70] Nagaoka K., Fujii K., Zhang H., Usuda K., Watanabe G., Ivshina M., Richter J.D. (2016). CPEB1 mediates epithelial-to-mesenchyme transition and breast cancer metastasis. Oncogene.

[71] Hägele S., Kühn U., Böning M., Katschinski D.M. (2009). Cytoplasmic polyadenylation-element-binding protein (CPEB)1 and 2 bind to the HIF-1alpha mRNA 3’-UTR and modulate HIF-1alpha protein expression. Biochem. J..

[72] Ortiz-Zapater E., Pineda D., Martínez-Bosch N., Fernández-Miranda G., Iglesias M., Alameda F., Moreno M., Eliscovich C., Eyras E., Real F.X. (2011). Key contribution of CPEB4-mediated translational control to cancer progression. Nat. Med..

[73] Pérez-Guijarro E., Karras P., Cifdaloz M., Martínez-Herranz R., Cañón E., Graña O., Horcajada-Reales C., Alonso-Curbelo D., Calvo T.G., Gómez-López G. (2016). Lineage-specific roles of the cytoplasmic polyadenylation factor CPEB4 in the regulation of melanoma drivers. Nat. Commun..

[74] Calderone V., Gallego J., Fernandez-Miranda G., Garcia-Pras E., Maillo C., Berzigotti A., Mejias M., Bava F.A., Angulo-Urarte A., Graupera G. (2016). Sequential functions of CPEB1 and CPEB4 regulate pathologic expression of vascular endothelial growth factor and angiogenesis in chronic liver disease. Gastroenterology.

[75] Degrauwe N., Suvà M.L., Janiszewska M., Riggi N., Stamenkovic I. (2016). IMPs: An RNA-binding brotein family that provides a link between stem cell maintenance in normal development and cancer. Genes Dev..

[76] Jonas K., Calin G.A., Pichler A. (2020). RNA-binding proteins as important regulators of long non-coding RNAs in cancer. Int. J. Mol. Sci..

[77] Nagaoka K., Udagawa T., Richter J.D. (2012). CPEB-mediated ZO-1 mRNA localization is required for epithelial tight-junction assembly and cell polarity. Nat. Commun..

[78] Farina K.L., Huttelmaier S., Musunuru K., Darnell R., Singer R.H. (2003). Two ZBP1 KH domains facilitate beta-actin mRNA localization granule formation, and cytoskeletal attachment. J. Cell Biol..

[79] Gu W., Katz Z., Wu B., Park H.Y., Li D., Lin S., Wells A.L., Singer R.H. (2012). Regulation of local expression of cell adhesion and motility-related mRNAs in breast cancer cells by IMP1/ZBP1. J. Cell Sci..

[80] Janiszewska M., Suvà M.L., Riggi N., Houtkooper R.H., Auwerx J., Clément-Schatlo V., Radovanovic I., Rheinbay E., Provero P., Stamenkovic I. (2012). Imp2 controls oxidative phosphorylation and is crucial for preserving glioblastoma cancer stem cells. Genes Dev..

[81] Noh J.H., Kim K.M., Abdelmohsen L., Yoon J.H., Panda A.C., Munk R., Kim J., Curtis J., Moad C.A., Wohler C.M. (2016). HuR and GRSF1 modulate the nuclear export and mitochondrial localization of the lncRNA RMRP. Genes Dev..

[82] Lubelsky Y., Ultsky I. (2018). Sequences enriched in Alu repeats drive nuclear localization of long RNAs in human cells. Nature.

[83] Nguyen T.M., Kabotyanski E.B., Reineke L.C., Shao J., Xiong F., Lee J.H., Dubrulle J., Johnson H., Stossi F., Tsoi P.S. (2020). The SINEB1 element in the long non-coding RNA Malat1 is necessary for TDP-43 proteostasis. Nucleic Acids Res..

[84] Garneau N.L., Wilusz J., Wilusz C.J. (2007). The highways and byways of mRNA decay. Nat. Rev. Mol. Cell Biol..

[85] Perron G., Jandaghi P., Solanki S., Safisamghabadi M., Storoz C., Karimzadeh M., Papadakis A.I., Arseneault M., Scelo G., Banks R.E. (2018). A general framework for interrogation of mRNA stability programs identifies RNA-binding proteins that govern cancer transcriptomes. Cell Rep..

[86] Moore A.E., Chenette D.M., Larkin L.C., Schneider R.J. (2014). Physiological networks and disease functions of RNA-binding protein AUF1. Wiley Interdiscip. Rev. RNA.

[87] Lin S., Wang W., Wilson G.M., Yang X., Brewer G., Holbrook N.J., Gorospe M. (2000). Down-regulation of cyclin D1 expression by prostaglandin A(2) Is mediated by enhanced cyclin D1 mRNA turnover. Mol. Cell Biol..

[88] Lal A., Mazan-Mamczarz K., Kawai T., Yang X., Martindale J.L., Gorospe M. (2004). Concurrent versus individual binding of HuR and AUF1 to common labile target mRNAs. EMBO J..

[89] Chang N., Yi J., Guo G., Liu X., Shang Y., Tong T., Cui Q., Ming Z., Gorospe M., Wang W. (2010). HuR uses AUF1 as a cofactor to promote p16INK4 mRNA decay. Mol. Cell Biol..

[90] Trojanowicz B., Brodauf L., Sekulla C., Lorenz K., Finke R., Dralle H., Hoang-Vu C. (2009). The role of AUF1 in thyroid carcinoma progression. Endocr. Relat. Cancer.

[91] Zucconi B.E., Wilson G.M. (2011). Modulation of neoplastic gene regulatory pathways by the RNAbinding factor AUF1. Front. Biosci..

[92] Yoon J.H., De S., Srikantan S., Abdelmohsen K., Grammatikakis I., Kim J., Kim J.M., Noh J.H., White E.J.F., Martindale J.L. (2014). PAR-CLIP analysis uncovers AUF1 impact on target RNA fate and genome integrity. Nat. Commun..

[93] Wang J., Guo Y., Chu H., Guan Y., Bi J., Wang B. (2013). Multiple functions of the RNA-binding protein HuR in cancer progression, treatment responses and prognosis. Int. J. Mol. Sci..

[94] Young L.E., Moore A.E., Sokol L., Meisner-Kober N., Dixon D.A. (2012). The mRNA stability factor HuR inhibits microRNA-16 targeting of COX-2. Mol. Cancer Res..

[95] Chai Y., Liu J., Zhang Z., Liu L. (2016). HuR-regulated lncRNA NEAT1 stability in tumorigenesis and progression of ovarian cancer. Cancer Med..

[96] Hu Y.P., Jin Y.P., Wu X.S., Yang Y., Li Y.S., Li H.F., Xiang S.S., Song X.L., Jiang L., Zhang Y.J. (2019). LNCRNA-HGBC stabilized by HuR promotes gallbladder cancer progression by regulating miR-502-3p/SET/AKT axis. Mol. Cancer.

[97] Kim J., Abdelmohsen K., Yang X., De S., Grammatikakis I., Noh H., Gorospe M. (2016). LncRNA OIP5-AS1/Cyrano sponges RNA-binding protein HuR. Nucleic Acids Res..

[98] Yoon J.H., Abdelmohsen K., Srikantan S., Yang X., Martindale J.L., De S., Huarte M., Zhang M., Becker K.G., Gorospe M. (2012). LincRNA-p21 suppresses target mRNA translation. Mol. Cell.

[99] Yoon J.H., Abdelmohsen K., Kim J., Yang X., Martindale J.L., Tominaga-Yamanaka K., White E.J., Orjalo A.V., Rinn J.L., Kreft S.G. (2014). Scaffold function of long non-coding RNA HOTAIR in protein ubiquitination. Nat. Commun..

[100] Fu M., Blackshear P.J. (2017). RNA-binding proteins in immune regulation: A focus on CCCH zinc finger proteins. Nat. Rev. Immunol..

[101] Sandler H., Kreth J., Timmers H.T.M., Stoecklin G. (2011). Not1 mediates recruitment of the deadenylase Caf1 to mRNAs targeted for degradation by tristetraprolin. Nucleic Acids Res..

[102] Fenger-Grøn M., Fillman C., Norrild B., Lykke-Andersen J. (2005). Multiple processing body factors and the ARE binding protein activate mRNA decapping. Mol. Cell.

[103] Park J.M., Lee T.H., Kang T.H. (2018). Roles of tristetraprolin in tumorigenesis. Int. J. Mol. Sci..

[104] Guo J., Qu H., Shan T., Chen Y., Chen Y., Xia J. (2018). Tristetraprolin overexpression in gastric cancer cells suppresses PD-L1 expression and inhibits tumor progression by enhancing antitumor immunity. Mol. Cells.

[105] Noubissi F.K., Elcheva I., Bhatia N., Shakoori A., Ougolkov A., Liu J., Minamoto T., Ross J., Fuchs S.Y., Spiegelman V.S. (2006). CRD-BP mediates stabilization of betaTrCP1 and c-myc mRNA in response to b-catenin signalling. Nature.

[106] Weidensdorfer D., Stöhr N., Baude A., Lederer M., Köhn M., Schierhorn A., Buchmeier S., Wahle E., Hüttelmaier S. (2009). Control of c-myc mRNA stability by IGF2BP1-associated cytoplasmic RNPs. RNA.

[107] Vikesaa J., Hansen T.V.O., Jønson L., Borup R., Wewer U.M., Christiansen J., Nielsen F.C. (2006). RNA-binding IMPs promote cell adhesion and invadopodia formation. EMBO J..

[108] Elcheva I., Goswami S., Noubissi F.K., Spiegelman V.S. (2009). CRD-BP protects the coding region of betaTrCP1 mRNA from miR-183-mediated degradation. Mol. Cell.

[109] Ye S., Song W., Xu X., Zhao X., Yang L. (2016). IGF2BP2 promotes colorectal cancer cell proliferation and survival through interfering with RAF-1 degradation by miR-195. FEBS Lett..

[110] Jønson L., Christiansen J., Hansen T.V.O., Vikeså J., Yamamoto Y., Nielsen F.C. (2014). IMP3 RNP safe houses prevent miRNA-directed HMGA2 mRNA decay in cancer and development. Cell Rep..

[111] Hämmerle M., Gutschner T., Uckelmann H., Ozgur S., Fiskin E., Gross M., Skawran B., Geffers R., Longerich T., Breuhahn K. (2013). Posttranscriptional destabilization of the liver-specific long noncoding RNA HULC by the IGF2 mRNA-binding protein 1 (IGF2BP1). Hepatology.

[112] Huang H., Weng H., Sun W., Qin X., Shi H., Wu H., Zhao B.S., Mesquita A., Liu C., Yuan C. (2018). Recognition of RNA N6-methyadenosine by IGF2BP proteins enhances mRNA stability and translation. Nat. Cell. Biol..

[113] Hellborg F., Qian W., Mendez-Vidal C., Asker C., Kost-Alimova M., Wilhelm M., Imreh S., Wiman K.G. (2001). Human wig-1, a p53 target gene that encodes a growth inhibitory zinc finger protein. Oncogene.

[114] Prahl M., Vilborg A., Palmberg C., Jörnvall H., Asker C., Wiman K.G. (2008). The p53 target protein Wig-1 binds hnRNP A2/B1 and RNA helicase A via RNA. FEBS Lett..

[115] Viborg A., Glahder J.A., Wilhelm M., Bersani C., Corcoran M., Mahmoudi S., Rosenstierne M., Grandér D., Farnebo M., Norrild B. (2009). The p53 target Wig-1 regulates p53 mRNA stability through an AU-rich element. Proc. Natl. Acad. Sci. USA.

[116] Kim B.C., Lee H.C., Lee J.J., Choi C.M., Kim D.K., Lee J.C., Ko Y.G., Lee J.S. (2012). Wig1 prevents cellular senescence by regulating p21 mRNA decay through control of RISC recruitment. EMBO J..

[117] Lee H.C., Jung S.H., Hwang H.J., Kang D., De S., Dudekula D.B., Martindale J.L., Park B., Park S.K., Lee E.K. (2017). Wig1 is crucial for AGO2-mediated ACOT7 mRNA silencing via miRNA-dependent and -independent mechanisms. Nucleic Acids Res..

[118] Truitt M.L., Ruggero D. (2017). New frontiers in translational control of the cancer genome. Nat. Rev. Cancer.

[119] Hsieh A.C., Ruggero D. (2010). Targeting eukaryotic translation initiation factor 4E (eIF4E) in cancer. Clin. Cancer Res..

[120] Mazan-Mamczarz K., Galbán S., López de Silanes I., Martindale J.L., Atasoy U., Keene J.D., Gorospe M. (2003). RNA-binding protein HuR enhances p53 translation in response to ultraviolet light irradiation. Proc. Natl. Acad. Sci. USA.

[121] Vo D.T., Abdelmohsen K., Martindale J.L., Qiao M., Tominaga K., Burton T.L., Gelfond J.A.L., Brenner A.J., Patel V., Trageser D. (2012). The oncogenic RNA-binding protein musashi1 is regulated by HuR via mRNA translation and stability in glioblastoma cells. Mol. Cancer Res..

[122] Galbán S., Kuwano Y., Pullmann R., Martindale M.L., Kim H.H., Lal A., Abdelmohsen K., Yang X., Dang Y., Liu J.O. (2008). RNA-binding proteins HuR and PTB promote the translation of hypoxia-inducible factor 1alpha. Mol. Cell Biol..

[123] Glorian V., Maillot G., Polès S., Iacovoni J.S., Favre G., Vagner S. (2011). HuR-dependent loading of miRNA RISC to the mRNA encoding the Ras-related small GTPase RhoB controls its translation during UV-induced apoptosis. Cell Death Differ..

[124] Lagadec C., Vlashi E., Frohnen P., Alhiyari Y., Chan M., Pajonk F. (2014). The RNA-binding protein Musashi-1 regulates proteasome subunit expression in breast cancer- and glioma-initiating cells. Stem Cells.

[125] Park S.-M., Gönen M., Vu L., Minuesa G., Tivnan P., Barlowe T.S., Taggart J., Lu Y., Deering R.P., Hacohen N. (2015). Musashi2 sustains the mixed-lineage leukemia-driven stem cell regulatory program. J. Clin. Investig..

[126] Niu J., Zhao X., Liu Q., Yang J. (2017). Knockdown of MSI1 inhibited the cell proliferation of human osteosarcoma cells by targeting p21 and p27. Oncol. Lett..

[127] Fox R.G., Lytle N.K., Jaquish D.V., Park F.D., Ito T., Bajaj J., Koechlein C.S., Zimdahl B., Yano M., Kopp J. (2016). Image-based detection and targeting of therapy resistance in pancreatic adenocarcinoma. Nature.

[128] Durie D., Lewis S.M., Liwak U., Kisilewicz M., Gorospe M., Holcik M. (2011). RNA-binding protein HuR mediates cytoprotection through stimulation of XIAP translation. Oncogene.

[129] Hussey G.S., Chaudhury A., Dawson A.E., Lindner D.J., Knudsen C.R., Wilce M.C.J., Merrick M.C.J., Howe P.H. (2011). Identification of an mRNP complex regulating tumorigenesis at the translational elongation step. Mol. Cell.

[130] Chaudhury A., Hussey G.S., Ray P.S., Jin G., Fox P.L., Howe P.H. (2010). TGF-beta-mediated phosphorylation of hnRNP E1 induces EMT via transcript-selective translational induction of Dab2 and ILEI. Nat. Cell Biol..

[131] Biyanee A., Ohnheiser J., Singh P., Klempnauer K.-H. (2015). A novel mechanism for the control of translation of specific mRNAs by tumor suppressor protein Pdcd4: Inhibition of translation elongation. Oncogene.

[132] Parsa N. (2012). Environmental Factors Inducing Human Cancers. Iran. J. Public Health.

[133] Lewandowska A.M., Rudzki M., Rudzki S., Lewandowski T., Laskowska B. (2019). Environmental risk factors for cancer. Ann. Agric. Environ. Med..

[134] Wang Z.-L., Li B., Luo Y.-X., Lin Q., Liu S.-R., Zhang X.-Q., Zhou H., Yang J.-H., Qu L.-H. (2018). Comprehensive genomic characterization of RNA-binding proteins across human cancers. Cell Rep..

[135] Gutschner T., Hämmerle M., Pazaitis N., Bley N., Fiskin E., Uckelmann H., Heim A., Groβ M., Hofmann N., Geffers R. (2014). Insulin-Like Growth Factor 2 mRNA-Binding Protein 1 (IGF2BP1) is an Important Protumorigenic Factor in Hepatocellular Carcinoma. Hepatology.

[136] Wang Z., Tong D., Han C., Zhao Z., Wang X., Jiang T., Li Q., Liu S., Chen L., Chen Y. (2019). Blockade of miR-3614 maturation by IGF2BP3 increases TRIM25 expression and promotes breast cancer cell proliferation. EBioMedicine.

[137] Mizutani R., Imamachi N., Suzuki Y., Yoshida H., Tochigi N., Oonishi T., Suzuki Y., Akimitsu N. (2016). Oncofetal protein IGF2BP3 facilitates the activity of proto-oncogene protein eIF4E through the destabilization of EIF4E-BP2 mRNA. Oncogene.

[138] Palanichamy J.K., Tran T.M., Howard J.M., Contreras J.R., Fernando T.R., Sterne-Weiler T., Katzman S., Toloue M., Yan W., Basso G. (2016). RNA-binding protein IGF2BP3 targeting of oncogenic transcripts promotes hematopoietic progenitor proliferation. J. Clin. Investig..

[139] Vargas T.R., Boudoukha S., Simon A., Souidi M., Cuvellier S., Pinna G., Polesskaya A. (2014). Post-transcriptional regulation of cyclins D1, D3 and G1 and proliferation of human cancer cells depend on IMP-3 nuclear localization. Oncogene.

[140] Bava F.A., Eliscovich C., Ferreira P.G., Minñana B., Ben-Dov C., Guigõ R., Juan Valcárcel J., Méndez R. (2013). CPEB1 coordinates alternative 3′-UTR formation with translational regulation. Nature.

[141] Kang H., Heo S., Shin J., Ji E., Tak H., Ahn S., Lee K., Lee E., Kim W. (2019). A miR-194/PTBP1/CCND3 axis regulates tumor growth in human hepatocellular carcinoma. J. Pathol..

[142] Liao B., Hu Y., Gary Brewer G. (2007). Competitive binding of AUF1 and TIAR to MYC mRNA controls its translation. Nat. Struct. Biol..

[143] Li F., Su M., Zhao H., Xie W., Cao S., Xu Y., Chen W., Wang L., Hou L., Tan W. (2019). HnRNP-F promotes cell proliferation by regulating TPX2 in bladder cancer. Am. J. Transl. Res..

[144] Guo X., Hartley R.S. (2006). HuR Contributes to Cyclin E1 Deregulation in MCF-7 Breast Cancer Cells. Cancer Res..

[145] Wang W., Caldwell M.C., Lin S., Furneaux H., Gorospe M. (2000). HuR regulates cyclin A and cyclin B1 mRNA stability during cell proliferation. EMBO Rep..

[146] Sommer G., Dittmann J., Kuehnert J., Reumann K., Schwartz P.E., Will H., Coulter B.L., Smith M.T., Heise T. (2011). The RNA-binding protein La contributes to cell proliferation and CCND1 expression. Oncogene.

[147] Tcherkezian J., Cargnello M., Romeo Y., Huttlin E.L., Lavoie G., Gygi S.P., Roux P.P. (2014). Proteomic analysis of cap-dependent translation identifies LARP1 as a key regulator of 5′ TOP mRNA translation. Genes Dev..

[148] Bechara E.G., Sebestyén E., Bernardis I., Eyras E., Valcárcel J. (2013). RBM5, 6, and 10 Differentially Regulate NUMB Alternative Splicing to Control Cancer Cell Proliferation. Mol. Cell.

[149] Feng C., Neumeister V., Ma W., Xu J., Lu L., Bordeaux J., Maihle N.J., Rimm D.L., Huang Y. (2012). Lin28 regulates HER2 and promotes malignancy through multiple mechanisms. Cell Cycle.

[150] Babic I., Jakymiw A., Fujita D.J. (2004). The RNA binding protein Sam68 is acetylated in tumor cell lines, and its acetylation correlates with enhanced RNA binding activity. Oncogene.

[151] Yang G., Fu1 H., Zhang J., Lu X., Yu F., Jin L., Bai L., Huang B., Shen L., Feng Y. (2010). RNA binding protein Quaking, a critical regulator of colon epithelial differentiation and a suppressor of colon cancer. Gastroenterology.

[152] Wong R. (2011). Apoptosis in cancer: From pathogenesis to treatment. J. Exp. Clin. Cancer Res..

[153] Abdelmohsen K., Lal A., Kim H., Gorospe M. (2007). Posttranscriptional Orchestration of an Anti-Apoptotic Program by HuR. Cell Cycle.

[154] Lal A., Kawai T., Yang X., Mazan-Mamczarz K., Gorospe M. (2005). Antiapoptotic function of RNA-binding protein HuR effected through prothymosin α. EMBO Rep..

[155] Wigington C.P., Jung J., Rye E.A., Belauret S.L., Philpot A.M., Feng Y., Santangelo P.J., Corbett A.H. (2015). Post-transcriptional Regulation of Programmed Cell Death (PDCD4) mRNA by the RNA-binding Proteins Human Antigen R (HuR) and T-cell Intracellular Antigen 1 (TIA1). J. Biol. Chem..

[156] Izquierdo J.M., Majós N., Bonnal S., Martínez C., Castelo R., Guigó R., Bilbao D., Valcárcel J. (2005). Regulation of Fas Alternative Splicing by Antagonistic Effects of TIA-1 and PTB on Exon Definition. Mol. Cell.

[157] Wendel H.S., Stanchina D., Fridman J.S., Malina A., Ray S., Kogan S., Cordon-Cardo C., Pelletier J., Lowe S.W. (2004). Survival signalling by Akt and eIF4E in oncogenesis and cancer therapy. Nature.

[158] Koso H., Yi H., Sheridan P., Miyano S., Ino Y., Todo T., Watanabe S. (2016). Identification of RNA-Binding Protein LARP4B as a Tumor Suppressor in Glioma. Cancer Res..

[159] Trotta R., Vignudelli T., Candini O., Intine R.V., Pecorari L., Guerzoni C., Santilli G., Byrom M.W., Goldoni S., Ford L.P. (2003). BCR/ABL activates mdm2 mRNA translation via the La antigen. Cancer Cell.

[160] Talwar S., Balasubramanian S., Sundaramurthy S., House R., Wilusz C.J., Kuppuswamy D., D’Silva N., Gillespie M.B., Hill E.G., Palanisamy V. (2013). Overexpression of RNA-binding protein CELF1 prevents apoptosis and destabilizes pro-apoptotic mRNAs in oral cancer cells. RNA Biol..

[161] Cheng F., Pan Y., Lu Y.M., Zhu L., Chen S. (2017). RNA-Binding Protein Dnd1 Promotes Breast Cancer Apoptosis by Stabilizing the Bim mRNA in a miR-221 Binding Site. Biomed. Res. Int..

[162] Sureban S.M., May R., George R.J., Dieckgraefe B.K., Mcleod H.L., Ramalingam S., Bishnupuri K.S., Natarajan G., Anant S., Houchen C.W. (2008). Knockdown of RNA binding protein Musashi-1 leads to tumor regression In Vivo. Gastroenterology.

[163] Gao X., Liu Z., Zhong M., Wu K., Zhang Y., Wang H., Zeng T. (2019). Knockdown of DNA/RNA-binding protein KIN17 promotes apoptosis of triple-negative breast cancer cells. Oncol. Lett..

[164] Jung J., Lee H., Cao B., Liao P., Zeng S.X., Lu H. (2020). RNA-binding motif protein 10 induces apoptosis and suppresses proliferation by activating p53. Oncogene.

[165] Lugano R., Ramachandran M., Dimberg A. (2020). Tumor angiogenesis: Causes, consequences, challenges and opportunities. Cell. Mol. Life Sci..

[166] Nabors L.B., Gillespie G.Y., Harkins L., King P.H. (2001). HuR, a RNA Stability Factor, Is Expressed in Malignant Brain Tumors and Binds to Adenine- and Uridine-rich Elements within the 3′ Untranslated Regions of Cytokine and Angiogenic Factor mRNAs. Cancer Res..

[167] Zadeh M., Amin E.M., Hoareau-Aveilla C., Domingo E., Symonds K.E., Yea X., Heesom K.J., Salmon A., D’Silva O., Betteridge K.B. (2015). Alternative splicing of TIA-1 in human colon cancer regulates VEGF isoform expression, angiogenesis, tumour growth and bevacizumab resistance. Mol. Oncol..

[168] Yang S.X., Hewitt S.M., Steinberg S.M., Liewehr D., Swain S.M. (2007). Expression Levels of eIF4E, VEGF, and Cyclin D1, and Correlation of eIF4E With VEGF and Cyclin D1 in Multi-Tumor Tissue Microarray. Oncol. Rep..

[169] Nathan C.A., Carter P., Liu L., Li B.D., Abreo F., Tudor A., Zimmer S.G., Benedetti A.D. (1997). Elevated Expression of eIF4E and FGF-2 Isoforms During Vascularization of Breast Carcinomas. Oncogene.

[170] Lee H.H., Son Y.J., Lee W.H., Park Y.W., Cha S.W., Cho W.J., Kim Y.M., Choi H.J., Choi D.H., Jung S.W. (2010). Tristetraprolin regulates expression of VEGF and tumorigenesis in human colon cancer. Int. J. Cancer.

[171] Shao R., Scully S.J., Yan W., Bentley B., Mueller J., Brown C., Bigelow C., Schwartz L.M. (2012). The novel lupus antigen related protein acheron enhances the development of human breast cancer. Int. J. Cancer..

[172] Lee M.Y., Lee J.-S. (2014). Exploiting tumor cell senescence in anticancer therapy. BMB Rep..

[173] Lee S.J., Lee J.-S. (2019). Cellular senescence: A promising strategy for cancer therapy. BMB Rep..

[174] Lee J.H., Jung M.S., Hong J.Y., Kim M.K., Chung I.K. (2018). Loss of RNA-binding protein HuR facilitates cellular senescence through posttranscriptional regulation of TIN2 mRNA. Nucleic Acids Res..

[175] Majumder M., House R., Palanisamy N., Qie S., Day T.A., Neskey D., Diehl J.A., Palanisamy V. (2016). RNA-Binding Protein FXR1 Regulates p21 and TERC RNA to Bypass p53-Mediated Cellular Senescence in OSCC. PLoS Genet..

[176] Pont A.R., Sadri N., Hsiao S.J., Smith S., Schneider R.J. (2012). mRNA decay factor AUF1 maintains normal aging, telomere maintenance and suppression of senescence by activation of telomerase transcription. Mol. Cell.

[177] Sanduja S., Kaza V., Dixon D.A. (2009). The mRNA decay factor tristetraprolin (TTP) induces senescence in human papillomavirus-transformed cervical cancer cells by targeting E6-AP ubiquitin ligase. Aging.

[178] Fregoso O.I., Das S., Akerman M., Krainer A.R. (2013). Splicing-Factor Oncoprotein SRSF1 Stabilizes p53 via RPL5 and Induces Cellular Senescence. Mol. Cell.

[179] Bebee T.W., Cieply B.W., Carstens R.P. (2014). Genome-wide Activities of RNA Binding Proteins That Regulate Cellular Changes in the Epithelial to Mesenchymal Transition (EMT). Adv. Exp. Med. Biol..

[180] Ueda J., Matsuda Y., Yamahatsu K., Uhida E., Naito Z., Korc M., Ishiwata T. (2014). Epithelial splicing regulatory protein 1 is a favorable prognostic factor in pancreatic cancer that attenuates pancreatic metastases. Oncogene.

[181] Lu H., Liu J., Liu S., Zeng J., Ding D., Carstens R.P., Cong Y., Xu X., Guo W. (2013). Exo70 Isoform Switching upon Epithelial-Mesenchymal Transition Mediates Cancer Cell Invasion. Dev. Cell.

[182] Tauler J., Zudaire E., Liu H., Shih J., Mulshine J.L. (2010). hnRNP A2/B1 Modulates Epithelial-Mesenchymal Transition in Lung Cancer Cell Lines. Cancer Res..

[183] Moran-Jones K., Grindlay J., Jones M., Smith R., Norman J.C. (2009). hnRNP A2 Regulates Alternative mRNA Splicing of TP53INP2 to Control Invasive Cell Migration. Cancer Res..

[184] Wang H., Vardy L.A., Tan C.P., Loo J.M., Guo K., Li J., Lim S.G., Zhou J., Chng W.J., Ng S.B. (2010). PCBP1 Suppresses the Translation of Metastasis-Associated PRL-3 Phosphatase. Cancer Cell.

[185] Howley B.V., Hussey G.S., Link L.A., Howe P.H. (2016). Translational regulation of inhibin βA by TGFβ via the RNA-binding protein hnRNP E1 enhances the invasiveness of epithelial-to-mesenchymal transitioned cells. Oncogene.

[186] Chen E.-B., Qin X., Peng K., Li Q., Tang C., Wei Y.-C., Yu S., Gan L., Liu T.-S. (2019). HnRNPR-CCNB1/CENPF axis contributes to gastric cancer proliferation and metastasis. Aging.

[187] Xu T., Zong Y., Peng L., Kong S., Zhou M., Zou J., Liu J., Mia R., Sun X., Li L. (2016). Overexpression of eIF4E in colorectal cancer patients is associated with liver metastasis. OncoTargets Ther..

[188] Gu W., Pan F., Singer R.H. (2009). Blocking -catenin binding to the ZBP1 promoter represses ZBP1 expression, leading to increased proliferation and migration of metastatic breast cancer cells. J. Cell Sci..

[189] Kim H.-Y., Thi H.T.H., Hong S.T. (2018). IMP2 and IMP3 cooperate to promote the metastasis of triple-negative breast cancer through destabilization of progesterone receptor. Cancer Lett..

[190] Petz M., Them N., Huber H., Beug H., Mikulits W. (2012). La enhances IRES-mediated translation of laminin B1 during malignant epithelial to mesenchymal transition. Nucleic Acids Res..

[191] Ji X., Lu H., Zhou Q., Luo K. (2014). LARP7 suppresses P-TEFb activity to inhibit breast cancer progression and metastasis. eLife.

[192] King C.E., Cuatrecasas M., Castells A., Sepulveda A.R., Lee J.-S., Rustgi A.K. (2011). LIN28B Promotes Colon Cancer Progression and Metastasis. Cancer Res..

[193] Richard S., Vogel G., Huot M.-É., Guo T., Muller W.J., Lukong K.E. (2008). Sam68 haploinsufficiency delays onset of mammary tumorigenesis and metastasis. Oncogene.

[194] Nakka K.K., Chaudhary N., Joshi S., Bhat J., Singh K., Chatterjee S., Malhotra R., De A., Santra M.K., Dilworth F.J. (2015). Nuclear matrix-associated protein SMAR1 regulates alternative splicing via HDAC6-mediated deacetylation of Sam68. Proc. Natl. Acad. Sci. USA.

[195] Vanharanta S., Marney C.B., Shu W., Valiente M., Zou Y., Mele A., Darnell R.B., Massagué J. (2014). Loss of the multifunctional RNA-binding protein RBM47 as a source of selectable metastatic traits in breast cancer. eLife.

[196] Mohibi S., Chen X., Zhang J. (2019). Cancer The ‘RBP’ eutics—RNA-Binding Proteins as Therapeutic Targets for Cancer. Pharmacol. Ther..

[197] Kentsis A., Topisirovic I., Culjkovic B., Shao L., Borden K.L.B. (2004). Ribavirin suppresses eIF4E-mediated oncogenic transformation by physical mimicry of the 7-methyl guanosine mRNA cap. Proc. Natl. Acad. Sci. USA.

[198] Li S., Jia Y., Jacobson B., McCauley J., Kratzke R., Bitterman P.B., Wagner C.R. (2013). Treatment of breast and lung cancer cells with a N-7 benzyl guanosine monophosphate tryptamine phosphoramidate pronucleotide (4Ei-1) results in chemosensitization to gemcitabine and induced eIF4E proteasomal degradation. Mol. Pharm..

[199] Moerke N.J., Aktas H., Chen H., Cantel S., Reibarkh M.Y., Fahmy A., Gross J.D., Degterev A., Yuan J., Chorev M. (2007). Small-Molecule Inhibition of the Interaction between the Translation Initiation Factors eIF4E and eIF4G. Cell.

[200] Cencic R., Hall D.R., Robert F., Du Y., Min J., Li L., Qui M., Lewis L., Kurtkaya S., Dingledine R. (2011). Reversing chemoresistance by small molecule inhibition of the translation initiation complex eIF4F. Proc. Natl. Acad. Sci. USA.

[201] Cao J., He L., Lin G., Hu C., Dong R., Zhang J., Zhu H., Hu Y., Wagner C.R., He Q. (2014). Cap-dependent translation initiation factor, eIF4E, is the target for Ouabain-mediated inhibition of HIF-1a. Biochem. Pharmacol..

[202] Peffley D.M., Sharma C., Hentosh P., Buechler R.D. (2007). Perillyl alcohol and genistein differentially regulate PKB/Akt and 4E-BP1 phosphorylation as well as eIF4E/eIF4G interactions in human tumor cells. Arch. Biochem. Biophys..

[203] Konicek B.W., Stephens J.R., McNulty A.M., Robichaud N., Peery R.B., Dumstorf C.A., Dowless M.S., Iversen P.W., Parsons S., Ellis K.E. (2011). Therapeutic Inhibition of MAP Kinase Interacting Kinase Blocks Eukaryotic Initiation Factor 4E Phosphorylation and Suppresses Outgrowth of Experimental Lung Metastases. Cancer Res..

[204] Ko S.Y., Guo H., Barengo N., Naora H. (2009). Inhibition of Ovarian Cancer Growth by a Tumor-Targeting Peptide That Binds Eukaryotic Translation Initiation Factor 4E. Clin. Cancer Res..

[205] Lucchesi C.A., Zhang J., Ma B., Chen M., Chen X. (2019). Disruption of the Rbm38-eIF4E Complex with a Synthetic Peptide Pep8 Increases p53 Expression. Cancer Res..

[206] Duffy A.G., Makarova-Rusher O.V., Ulhannan S.V., Rahma O.E., Fioravanti S., Walker M., Abdullah S., Raffeld M., Anderson V., Abi-Jaoudeh N. (2016). Modulation of tumor eIF4E by antisense inhibition: A phase I/II translational clinical trial of ISIS 183750—An antisense oligonucleotide against eIF4E—In combination with irinotecan in solid tumors and irinotecan-refractory colorectal cancer. Int. J. Cancer.

[207] Hong D.S., Kurzrock R., Oh Y., Wheler J., Naing A., Brail L., Callies S., André V., Kadam S.K., Nasir A. (2011). A Phase 1 Dose Escalation, Pharmacokinetic, and Pharmacodynamic Evaluation of eIF-4E Antisense Oligonucleotide LY2275796 in Patients with Advanced Cancer. Clin. Cancer Res..

[208] Dong K., Wang R., Wang X., Lin F., Shen J.-J., Gao P., Zhang H.-Z. (2009). Tumor-specific RNAi targeting eIF4E suppresses tumor growth, induces apoptosis and enhances cisplatin cytotoxicity in human breast carcinoma cells. Breast Cancer Res. Treat..

[209] Lang M., Berry D., Passecker K., Mesteri I., Bhuju S., Ebner F., Sedlyarov V., Evstatiev R., Dammann K., Loy A. (2017). HuR small-molecule inhibitor elicits differential effects in adenomatosis polyposis and colorectal carcinogenesis. Cancer Res..

[210] Lal P., Cerofolini L., D’Agostino V.G., Zucal C., Fuccio C., Bonomo I., Dassi E., Giuntini S., Maio D.D., Vishwakarma V. (2017). Regulation of HuR structure and function by dihydrotanshinone-I. Nucleic Acids Res..

[211] Filippova N., Yang X., Ananthan S., Sorochinsky A., Hackney J.R., Gentry Z., Bae S., King P., Nabors L.B. (2017). Hu antigen R (HuR) multimerization contributes to glioma disease progression. J. Biol. Chem..

[212] Kaur K., Wu X., Fields J.K., Johnson D.K., Lan L., Pratt M., Somoza A.D., Wang C.C.C., Karanicolas J., Oakley B.R. (2017). The fungal natural product azaphilone-9 binds to HuR and inhibits HuR-RNA interaction in vitro. PLoS ONE.

[213] Muralidharan R., Mehta M., Ahmed R., Roy S., Xu L., Aubé J., Chen A., Zhao Y.D., Herman T., Ramesh R. (2017). HuR-targeted small molecule inhibitor exhibits cytotoxicity towards human lung cancer cells. Sci. Rep..

[214] Allegri L., Baldan F., Roy S., Aubé J., Russo D., Filetti S., Damante G. (2019). The HuR CMLD-2 inhibitor exhibits antitumor effects via MAD2 downregulation in thyroid cancer cells. Sci. Rep..

[215] Amreddy N., Babu A., Paneerselvam J., Srivastava A., Muralidharna R., Chen A., Zhao Y.D., Munshi A., Ramesh R. (2018). Chemo-biologic combinatorial drug delivery using folate receptor-targeted dendrimer nanoparticles for lung cancer treatment. Nanomedicine.

[216] Muralidharan R., Babu A., Amreddy N., Srivastava A., Chen A., Zhao Y.D., Kompella U.B., Munshi A., Ramesh R. (2017). Tumor-targeted Nanoparticle Delivery of HuR siRNA Inhibits Lung Tumor Growth In Vitro and In Vivo By Disrupting the Oncogenic Activity of the RNA-binding Protein HuR. Mol. Cancer Ther..

[217] Clingman C.C., Deveau L.M., Hay S.A., Genga R.M., Shandilya S.M.D., Massi F., Ryder S.P. (2014). Allosteric inhibition of a stem cell RNA-binding protein by an intermediary metabolite. eLife.

[218] Lan L., Appelman C., Smith A.R., Yu J., Larsen S., Marquez R.T., Liu H., Wu X., Gao P., Roy A. (2015). Natural product (L)-gossypol inhibits colon cancer cell growth by targeting RNA-binding protein Musashi-1. Mol. Oncol..

[219] Minuesa G., Albanese S.K., Xie W., Kazansky Y., Worroll D., Chow A., Schurer A., Park S.M., Rotsides C.Z., Taggart J. (2019). Small-molecule targeting of MUSASHI RNA-binding activity in acute myeloid leukemia. Nat. Commun..

[220] Chen H., Liu J., Wang H., Cheng Q., Zhou C., Chen X., Ye F. (2019). Inhibition of RNA-Binding Protein Musashi-1 Suppresses Malignant Properties and Reverses Paclitaxel Resistance in Ovarian Carcinoma. J. Cancer.

[221] Roos M., Pradére U., Ngondo R.P., Behera A., Allegrini S., Civenni G., Zagalak J.A., Marchand J.-R., Menzi M., Towbin H. (2016). A Small-Molecule Inhibitor of Lin28. ACS Chem. Biol..

[222] Wang L., Rowe R.G., Jaimes A., Yu C., Nam Y., Pearson D.S., Zhang J., Xie X., Marion W., Heffron G.J. (2018). Small-molecule inhibitors disrupt let-7 oligouridylation and release the selective blockade of let-7 processing by LIN28. Cell Rep..

[223] Marqus S., Pirogova E., Piva T.J. (2017). Evaluation of the use of therapeutic peptides for cancer treatment. J. Biomed. Sci..

[224] Watts J.K., Corey D.R. (2012). Gene silencing by siRNAs and antisense oligonucleotides in the laboratory and the clinic. J. Pathol..

[225] Chi X., Gatti P., Papoian T. (2017). Safety of antisense oligonucleotide and siRNA-based therapeutics. Drug Discov. Today.

[226] Burdelski C., Jakani-karimi N., Jacobsen F., Möller-Koop C., Minner S., Simon R., Sauter G., Steurer S., Clauditz T.S., Wilczak W. (2018). IMP3 overexpression occurs in various important cancer types and is linked to aggressive tumor features: A tissue microarray study on 8877 human cancers and normal tissues. Oncol. Rep..

[227] Morimatsu K., Aishima S., Yamamoto H., Hayashi A., Nakata K., Oda Y., Shindo K., Fujino M., Tanaka M., Oda Y. (2013). Insulin-like growth factor II messenger RNA–binding protein-3 is a valuable diagnostic and prognostic marker of intraductal papillary mucinous neoplasm. Hum. Pathol..

[228] Heinonen M., Bono P., Narko K., Chang S.-H., Lundin J., Joensuu H., Furneaux H., Hla T., Haglund C., Ristimäki A. (2005). Cytoplasmic HuR Expression Is a Prognostic Factor in Invasive Ductal Breast Carcinoma. Cancer Res..

[229] Huang H., Han Y., Zhang C., Wu J., Feng J., Qu L., Shou C. (2016). HNRNPC as a candidate biomarker for chemoresistance in gastric cancer. Tumor Biol..

[230] Wang K., Li L., Fu L., Yuan Y., Dai H., Zhu T., Zhou Y., Yuan F. (2019). Integrated Bioinformatics Analysis the Function of RNA Binding Proteins (RBPs) and Their Prognostic Value in Breast Cancer. Front. Pharmacol..

[231] Wei L., Gao L.-N., Song P.-P., You C.-G. (2020). Development and validation of a RNA binding protein-associated prognostic model for lung adenocarcinoma. Aging.

[232] Kaczmarek J.C., Kowalski P.S., Anderson D.G. (2017). Advances in the delivery of RNA therapeutics: From concept to clinical reality. Genome Med..

